# Tumor-induced STAT3 activation in monocytic myeloid-derived suppressor cells enhances stemness and mesenchymal properties in human pancreatic cancer

**DOI:** 10.1007/s00262-014-1527-x

**Published:** 2014-03-21

**Authors:** Roheena Z. Panni, Dominic E. Sanford, Brian A. Belt, Jonathan B. Mitchem, Lori A. Worley, Brian D. Goetz, Pinku Mukherjee, Andrea Wang-Gillam, Daniel C. Link, David G. DeNardo, S. Peter Goedegebuure, David C. Linehan

**Affiliations:** 1 Department of Surgery, Washington University School of Medicine, 660 South Euclid Ave., Box 8109, St. Louis, MO 63110 USA; 2 Division of Oncology, Department of Medicine, Washington University School of Medicine, St. Louis, MO USA; 3 Alvin J. Siteman Cancer Center, St. Louis, MO USA; 4 Department of Biology, University of North Carolina at Charlotte, Charlotte, NC USA

**Keywords:** Monocytic MDSC, Stem cell, Epithelial to mesenchymal transition, Pancreatic cancer, STAT3 activity

## Abstract

Pancreatic cancer (PC) mobilizes myeloid cells from the bone marrow to the tumor where they promote tumor growth and proliferation. Cancer stem cells (CSCs) are a population of tumor cells that are responsible for tumor initiation. Aldehyde dehydrogenase-1 activity in PC identifies CSCs, and its activity has been correlated with poor overall prognosis in human PC. Myeloid cells have been shown to impact tumor stemness, but the impact of immunosuppressive tumor-infiltrating granulocytic and monocytic myeloid-derived suppressor cells (Mo-MDSC) on ALDH1^Bright^ CSCs and epithelial to mesenchymal transition is not well understood. In this study, we demonstrate that Mo-MDSC (CD11b^+^/Gr1^+^/Ly6G^−^/Ly6C^hi^) significantly increase the frequency of ALDH1^Bright^ CSCs in a mouse model of PC. Additionally, there was significant upregulation of genes associated with epithelial to mesenchymal transition. We also found that human PC converts CD14^+^ peripheral blood monocytes into Mo-MDSC (CD14^+^/HLA-DR^low/−^) in vitro, and this transformation is dependent on the activation of the STAT3 pathway. In turn, these Mo-MDSC increase the frequency of ALDH1^Bright^ CSCs and promote mesenchymal features of tumor cells. Finally, blockade of STAT3 activation reversed the increase in ALDH1^Bright^ CSCs. These data suggest that the PC tumor microenvironment transforms monocytes to Mo-MDSC by STAT3 activation, and these cells increase the frequency of ALDH1^Bright^ CSCs. Therefore, targeting STAT3 activation may be an effective therapeutic strategy in targeting CSCs in PC.

**Electronic supplementary material:**

The online version of this article (doi:10.1007/s00262-014-1527-x) contains supplementary material, which is available to authorized users.

## Introduction

Pancreatic cancer (PC) is a highly aggressive malignancy which is characterized by early metastasis and chemotherapeutic resistance [1]. PC has a uniquely dense stroma with abundant leukocytes, which are predominantly myeloid cells. These myeloid cells are heterogeneous; primarily comprised of macrophages and monocytic and granulocytic myeloid-derived suppressor cells (Mo- and G-MDSC, respectively) [2]. These cells are produced in the bone marrow, are actively recruited to the tumor microenvironment by tumor-derived chemokines, and promote tumor growth and spread through various mechanisms [3–5].

Their clinical importance is demonstrated by the finding that in a number of cancer types including PC, increased prevalence of myeloid cells in the tumor microenvironment is an independent prognostic factor for survival [6]. Tumor-associated myeloid cells have been shown to correlate with disease stage, resectability, and survival in PC [7, 8]. Typical of tumor-infiltrating myeloid cells is their ability to suppress antitumor immunity [9]. In addition, they can also directly promote tumor cell proliferation, invasion and thus facilitate metastasis, and therapeutic resistance [7, 10].

Cancer stem cells (CSCs) are an important subpopulation of tumor cells which are capable of tumor initiation and are resistant to chemotherapy [11]. In PC, CSCs were first identified as CD24^+^/CD44^+^/ESA^+^ cells [12]. Aldehyde dehydrogenase-1 (ALDH1) is a relatively new marker which is an intracellular detoxifying enzyme originally identified as a phenotypic marker for hematopoietic stem cells [13, 14]. We and others have previously demonstrated that ALDH1 is also an important marker of CSCs in PC [15, 16]. In fact, patients having tumors with increased ALDH1^Bright^ CSCs have decreased progression free and overall survival [16]. It is not entirely clear how levels of ALDH1^Bright^ CSCs are regulated, but there is mounting evidence for a dynamic interplay between the stroma and tumor cells which promotes epithelial to mesenchymal transition (EMT) and tumor stemness [17, 18].

The role of mature tumor-associated macrophages (TAMs) in promoting stemness in a murine model of PC has been demonstrated by Mitchem et al. However, the role of Mo-MDSC in PC is not well understood. In this study, G-MDSC and Mo-MDSC from both mice (G-MDSC: CD11b^+^/Gr1^+^/Ly6G^+^/Ly6C^mid^, Mo-MDSC: CD11b^+^/Gr1^+^/Ly6G^−^/Ly6C^hi^) and humans (G-MDSC: Lin^−^/CD11b^+^/CD33^+^/CD15^+^, Mo-MDSC: Lin^−^/CD11b^+^/CD33^+^/CD14^+^/HLA-DR^low/−^) [19, 20] were assessed for their impact on ALDH1^Bright^ CSC in mouse and human PC. We demonstrate that Mo-MDSC-infiltrating PC tumors promote the ALDH1^Bright^ CSC population by activation of STAT3. We also show that cancer stem cell promoting activity can be blocked by inhibition of the STAT3 signaling pathway.

## Materials and methods

### Bone marrow samples from pancreatic cancer patients and healthy controls and collection of PC tissue from patients

Blood, bone marrow samples (*N* = 16), and tumor samples (*N* = 11) were collected from patients with resectable PC at Barnes-Jewish Hospital (St. Louis, MO, USA). These patients received no chemotherapy or radiation therapy prior to surgery. Informed consent was obtained on all patients in accordance with Institutional Review Board (IRB)-approved protocol. Normal bone marrow was obtained from age-matched healthy cancer free volunteers (*N* = 7). Bone marrow samples were collected in vacuum tubes containing lithium heparin (BD Vacutainer), and mononuclear cells were isolated by Ficoll density centrifugation. Normal pancreas tissue was collected from patients (*N* = 4) who were eligible for organ donation and had no malignant disease. Paraffin slides of normal human pancreas tissue (*N* = 5) were obtained from Abcam, GeneTex, IHC world, and Imgenex.

### Pancreatic cancer tissue microarray survival analysis

After obtaining IRB approval, tissue microarray (TMA) studies were conducted on a cohort of 60 previously untreated patients with pancreatic ductal adenocarcinoma who underwent pancreaticoduodenectomy at Barnes-Jewish Hospital. Patients did not receive neo-adjuvant therapy and were typically treated with adjuvant chemotherapy. To construct the TMA, well-defined areas of tumor were demarcated and punched (1 mm diameter) from paraffin-embedded tumor blocks. An Aperio Scan-Scope XT Slide Scanner (Aperio Technologies) system was used to acquire digital images using a 20× objective. A tumor-specific nuclear algorithm (IHC-MARK) developed in-house [15] was modified to quantify CD14, CD8, and ALDH1 expression.

### STAT3 inhibitor

STATTIC (STAT3 inhibitor V) was obtained from Calbiochem/EMD and used at doses less than the reported IC50 (<20 µM) in vitro according to manufacturer instructions [21].

### Cell lines

KCM and KCKO were a kind gift from Dr. Mukherjee [22], Panc-1 and BxPC3 were purchased from ATCC, and Pan02 [23], from the Division of Cancer Treatment Tumor Repository (NCI-Frederick Cancer Research and Development Center, Bethesda, MD).

### Animal husbandry

GCSFR^−/−^ mice were a kind gift from Daniel C. Link (Internal Medicine/Division of Oncology). C57BL/6 and NU/J mice were purchased from Jackson Laboratories. Mice were maintained within the Washington University Laboratory for Animal Care barrier facility and all studies involving animals were approved by the Washington University School of Medicine Institutional Animal Studies Committee.

### Orthotopic model and preclinical animal cohorts

Six- to eight-week-old GCSFR^−/−^ and wild-type (WT) mice were injected in the tail of the pancreas with 1 × 10^5^ murine tumor cells suspended in 50 μl of PBS and Matrigel (mixed 1:1). Mice were sacrificed at 31 days post-injection and peripheral blood, bone marrow, and tumors were collected. The bone marrow was extracted from femurs via high-speed centrifugation, and blood samples were collected in heparinized capillary tubes from retro-orbital sinus. Blood and bone marrow were washed once with sterile phosphate buffered saline (PBS) and subjected to red blood cell (RBC) lysis, according to manufacturer’s instructions (RBC lysis buffer, Biolegend). We measured gross wet weight of orthotopic tumors. The tumors were minced into 2–3-mm-size pieces, mechanically dissociated using the Miltenyi Gentle-MACS tissue dissociator and digested in enzyme buffer that contained 1 mg/ml collagenase, 2.5 U/ml hyaluronidase and 0.1 mg/ml of DNAse for 30 min at 37 °C. Digestion mixtures were quenched by adding DMEM with 10 % FBS and filtered through 70-μm nylon strainers (BD falcon) and were counted. Pieces of tumor were also snap frozen in liquid nitrogen. Mice were injected with 2 × 10^5^ tumor cells in 100 μl PBS subcutaneously, and tumor volume was calculated at specific time intervals by caliper measurements. We used 7–10 mice per group, and all these experiments were repeated in triplicate. All experiments were carried out according to animal studies committee guidelines.

### Flow cytometry

Human and mouse single cell suspensions were blocked with TruStain FcX or anti CD16/32 antibody, respectively (Biolegend) and stained with fluorescent antibodies using standard protocols for flow cytometry. To stain ALDH^Bright^ cells, the Aldefluor assay was performed according to the manufacturer’s protocol (Stem Cell Technologies). For intracellular staining of cells, we used permeabilization buffer (eBioscience). We used CD45 (AF700; Biolegend, HI30), CD11b (AF488 and Pacific Blue; Biolegend, ICRF44), CD115 (PE; Biolegend, 9-4D21E4), CD14 (APC/Cy7; Biolegend, M5E2), CD15 (Pacific Blue; Biolegend, W6D3), CD68 (APC; Biolegend, CD68), HLA-DR (PE/Cy7; BIolegend, L243), CD4 (PerCP/Cy5.5; eBioscience, RPA-T4), CD8 (Pacific Blue; Biolegend, SK1), EpCAM (Pac Blue; Biolegend, 9C4), CD24(PE/Cy7; Biolegend, ML5), CD44 (APC/Cy7; Biolegend, IM7), and PPI (Biolegend) for human tumors. For mice, we used CD45 (APC/Cy7; Biolegend, 30-F11), CD11b (APC; Biolegend, M1/70), Ly6C (PE/Cy7; Biolegend HK1.4), Ly6G (Pacific Blue; Biolegend, 1A8), Gr-1 (AF700; Biolegend, RB6-865), F4/80 (PerCpCy5.5; Biolegend, BM8), I-A/I-E (AF488; Biolegend, M5/114.15.2), CD4 (AF700; Biolegend, GK1.5), CD8a (PE; Biolegend, 53-6.7), EpCAM (PE/Cy7; Biolegend, G8.8), CD24 (PerCP/Cy5.5; Biolegend, M1/69), CD44 (APC/Cy7; Biolegend, IM7), and PPI(Biolegend). Data acquisition and analysis were performed using an LSRII system (BD Biosciences), Aria II High Speed Cell Sorter (BD Biosciences) for fluorescence-activated cell sorting (FACS) and FlowJo version 7.6.5 software (Tree Star).

### RNA isolation and RT-PCR

Snap-frozen tissue from mouse and human was homogenized in TRIzol Reagent (Life Technologies), and total RNA was isolated according to the manufacturer’s guidelines. RNA samples were treated with DNAse (Promega), and the RNA was extracted with the RNeasy Mini Kit using the manufacturer’s instructions for RNA clean up (Qiagen). RNA was reverse transcribed into cDNA using the high capacity RNA-to-cDNA kit (Life Technologies), and real-time PCR reactions were prepared by mixing cDNA with Taqman FAST universal PCR Master Mix and pre-designed Taqman Gene Expression assays (Life Technologies). Real-time PCR was performed on a 7500 FAST Thermal cycler (Applied Biosystems). Target gene expression was normalized to GAPDH, HPRT, or β-actin. Target gene expression was determined using the comparative CT (ΔΔ*C*_T_) method with 7500 software for 7500 RT-PCR system version 2.0.6 (Applied Biosystems).

### CD8 suppression assays

PBMCs were isolated from healthy volunteers by Ficoll density centrifugation. CD14^+^ cells were isolated from the PBMCs of healthy volunteers with the EasySep^®^ Human CD14 Positive Selection Kit according to the manufacturer’s instructions (Stemcell Technologies™). CD14^+^ cells were cocultured with BXPC3 or Panc-1 tumor cells in a transwell plate to prevent cell to cell contact between the two populations. The CD14^+^ cells were mixed 1:1 with autologous PBMCs labeled with 10 μM CFSE (Life Technologies) in 96-well round-bottom plates (Corning) coated with 1 μg/ml LEAF™ purified anti-human CD3 (Biolegend, clone OKT3) in complete media [RPMI containing 2 mM glutamine, 100 U/ml penicillin, 100 μg/ml streptomycin, 1 mM sodium pyruvate (Life Technologies), and 10 % fetal bovine calf serum (Hyclone)] supplemented with 100 U/ml of human interleukin 2 (National Institute of Health). Cell cultures were harvested after 72 h at 37 °C, and the CFSE dilution of the CD4^+^ and CD8^+^ T cell fractions was analyzed by flow cytometry.

In order to test the immunosuppressive properties of mouse Mo-MDSC, total mouse bone marrow was isolated from the femur and tibia of WT and G-CSFR^−/−^ mice by high-speed centrifugation. Bone marrow was subjected to RBC lysis in Tris lysing buffer (144 mM NH_4_Cl, 17 mM Tris–HCl, pH 7.2), and CD11b^+^ cells were isolated using the mouse CD11b positive selection kit according to the manufacturer’s instructions (Stemcell Technologies). CD11b^+^ cells were converted into Mo-MDSC by incubating in Pan02-conditioned medium for 72 h at 37 °C in 6-well flat-bottom plates. Tumor-conditioned media was prepared by incubating Pan02 in complete media in T75 flasks for 72 h at 37 °C followed by 0.2 μm filtration. Single cell suspensions of mouse splenocytes were prepared by crushing the spleens of WT mice in PBS and filtering the suspensions over 40 μm filters. Single cell suspensions were subjected to RBC lysis and splenocytes were labeled with CFSE as described above. Labeled splenocytes were cocultured in complete media supplemented with 50 μM 2-mercaptoethanol in 96-well round-bottom plates (Corning) coated with 0.5 μg/ml LEAF™ purified anti-mouse CD3 (Biolegend, clone 145-2C11) and varying concentrations of CD11b^+^ cells previously treated with complete media alone or tumor-conditioned media. In some wells, arginase-1 activity was inhibited by adding nor-NOHA (Calbiochem) at a final concentration of 0.5 mM. Cell cultures were harvested after 72 h at 37 °C, and the CFSE dilution of the CD4^+^ and CD8^+^ T cell fractions was analyzed by flow cytometry.

### Immunofluorescence

Formalin-fixed paraffin-embedded tissue blocks of archived human pancreatic ductal adenocarcinoma were cut at 5 μm using a Leica RM2235 microtome. Cut tissue sections were then floated onto SuperFrost Plus slides (Fisher Scientific) in a 42 °C water bath and dried overnight. Normal pancreas tissue previously cut and mounted on glass slides was purchased from Imgenex. Tissue sections were heated for 30 min at 65 °C and deparaffinized in three changes of xylene. The sections were then rehydrated in serial changes in decreasing ethanol in deionized water (100, 95, 70, 50 %, then deionized H_2_O) and rinsed in PBS. Tissue sections were submerged in citrate buffer (10 mM sodium citrate, 0.05 % Tween 20, pH 6) or Target Retrieval Solution (DAKO, pH 9), and antigen retrieval was performed in a decloaking chamber (Biocare Medical). The sections were washed in running deionized water and rinsed in PBS. Tissue sections were then blocked for 30 min at room temperature in Serum-Free Protein Block (DAKO) and stained with primary antibodies diluted in Antibody Diluent (DAKO) overnight at 4 °C. The following primary antibodies were used in this study: mouse anti-ALDH1, clone 44/ALDH (BD, 1:100); rabbit anti-CD11b, clone M1/70 (Abcam, 1:100); mouse anti-CD14 (Thermo Fisher Scientific Inc., 1:20); mouse anti-CD15, clone MY-1 (Abcam, 1:10); mouse anti-keratin 7, clone OV-TL 12/30 (Thermo Fisher Scientific, 1:50); and mouse anti-phospho-Stat3, clone D3A7 (Cell Signaling Technology, 1:400). Tissue sections were washed in PBS then stained with Alexa 488 goat anti-mouse IgG and Alexa 555 goat anti-rabbit IgG secondary antibodies (Life Technologies) diluted 1:200 in Antibody Diluent for 30 min at room temperature. TO-PRO-3 iodide (Life Technologies) was added to the secondary antibody cocktail at a final concentration of 1 μM to visualize cellular nuclei. The tissue sections were washed in PBS, and then, the excess PBS was blotted off and sections were coverslipped with Vectashield Hard Mounting Media (Vector Labs). Confocal images were acquired on an Axiovert 100M microscope equipped with a LSM 510 META Confocal Laser Scanning Microscope system (Zeiss).

### Western blotting

CD14^+^ cells previously cocultured with Panc-1 tumor cells (separated by a transwell insert) or cultured in complete media alone. After coculture, CD14^+^ cells were separated and washed with 1× PBS and lysed in RIPA buffer containing a protease inhibitor (Roche) and phosphatase inhibitor (Sigma) cocktails. Cell lysates were sonicated for 10 s then centrifuged at 4 °C for 10 min at 14,000×*g*. The supernatant was removed and protein concentration was determined using a BCA Protein Assay kit (Lamda Biotech, Inc.,). Twenty microgram of total protein was prepared for denaturing gel electrophoresis under non-reducing conditions in NuPAGE^®^ LDS buffer according to the manufacturer’s instructions and loaded onto NuPAGE^®^ 4–12 % Bis–Tris gels in an XCell SureLock Mini-Cell containing 1× NuPAGE^®^ MOPS SDS running buffer (Life Technologies). Gels were run at 200 V for 50 min and then blotted onto PVDF membranes in an XCell Surelock Mini-Cell by wet transfer with 1× NuPAGE transfer buffer (Life Technologies). After transfer, the blots were rinsed in washing buffer (TBST: 20 mM Tris base, 137 mM NaCl, 0.1 % Tween 20, pH 7.6) and blocked in blocking buffer (5 % non-fat dry milk in TBST) for 1 h at room temperature. The membranes were washed in TBST and incubated in rabbit anti-phospho-STAT3 (Cell Signaling Technologies, clone D3A7; 1:2,000) or goat anti-actin (Santa Cruz Biotechnology, Inc., 1:200) primary antibodies diluted in TBST with 5 % BSA overnight at 4 °C. Membranes were washed in TBST and incubated in goat anti-rabbit IgG-HRP (Santa Cruz Biotechnology, Inc., 1:2,000) or donkey anti-goat IgG-HRP (Santa Cruz Biotechnology, Inc., 1:5,000) diluted in blocking buffer for 1 h at room temperature. The membranes were washed in TBST and protein bands were visualized with SuperSignal West Dura Extended Duration Substrate (Thermo Fisher Scientific). Blot images were acquired with a ChemiDoc XRS+ imaging system (Bio-Rad).

### Invasion assays

BD BioCoat Matrigel Invasion chambers (Catalog# 354480) were used to study the invasion of tumor cell lines cocultured with and without CD11b^+^ or CD14^+^ cells. BD BioCoat chambers (24-well) were rehydrated using bicarbonate-based culture medium. CD11b^+^ and CD14^+^ cells were isolated from mice and humans, respectively, as described above and were plated in the bottom of 6-well chambers. The transwell inserts were placed in the chamber after 6 h of plating cells. Single cell suspensions of tumors (BxPC3, Panc-1, KCKO, KCM) were made in culture medium, and 2.5 × 10^4^ tumor cells (in 0.5 ml of culture medium) were added on top of the transwell insert to assess their ability to migrate/invade (downward) through the Matrigel-coated membrane. The cells were incubated in a humidified tissue culture incubator at 37 °C for 22 h. The non-invading cells were removed from the upper surface of the membrane using a Q-tip. The cells that invaded through the Matrigel-coated membrane were then fixed using 100 % methanol. After fixation, the cells that invaded through Matrigel membrane were stained using 1 % toluidine blue (Sigma, Catalog# 198161). The inserts were washed with diH_2_O, air-dried, and membranes were removed from the inserts. The membranes were placed on a microscope slide with a small drop of immersion oil, and a cover slip was placed on top. Images of invaded cells were acquired under microscope at 40× magnification using SPOT RT digital camera and software. Cells were counted in six different fields of triplicate membranes.

### Human and mouse bone marrow colony-forming unit (CFU) assay

Methylcellulose-based culture (MC) media (MethoCult, StemCell Technologies Catalog# 03001) was thawed under refrigeration (2–8 °C) overnight. Bone marrow cells were isolated from both human and mouse and were resuspended in PBS with 2 % FBS at 2 × 10^6^ cells/ml. The cell suspension was added to Methocult medium at a 1:10 ratio to maintain correct medium viscosity. Three milliliter of the suspension was drawn and plated in a 35 mm low-adherence petri dish with a loose fitting lid. The petri dishes were placed in a large petri dish and incubated in a humidified tissue culture incubator at 37 °C for 7 days. Granulocyte and macrophage colony-forming units (CFU-GM) were counted at 25× to 50× and a higher magnification was used to confirm CFU type. The comparison was made between PC patient bone marrow and healthy donor bone marrow. For mouse studies, the comparison was made between GCSFR^−/−^ and WT bone marrow.

### Coculture experiments with transwell insert

All cocultures were performed using transwell inserts (BD Falcon Catalog# 353090) so the tumor cells were separated from myeloid cells. CD14^+^ cells or CD11b^+^ cells suspended in cell culture medium were plated on the 6-well cell culture plate (costar# 3516). The transwell inserts were gently transferred into the previously filled wells (with CD11b^+^ or CD 14^+^ cells), and tumor cell suspension was added on top of the transwell insert. Tumor cells were isolated after coculture by removing the transwell inserts. CD14^+^ cells from the bottom were separately harvested. Isolated tumor cells and CD14^+^ cells were washed, and flow cytometry RT-PCR, and Western blotting were performed.

### Statistical analysis

Graphpad prism V (Graphpad Software Inc.,) was used for statistical analysis. For nonparametric data Mann–Whitney test and for parametric data unpaired *t* test was used *p* < 0.05 was considered statistically significant.

## Results

### Pancreatic cancer is characterized by abundant granulocytic and monocytic myeloid cells as well as ALDH1^Bright^ cancer stem cells

**Fig. 1:**
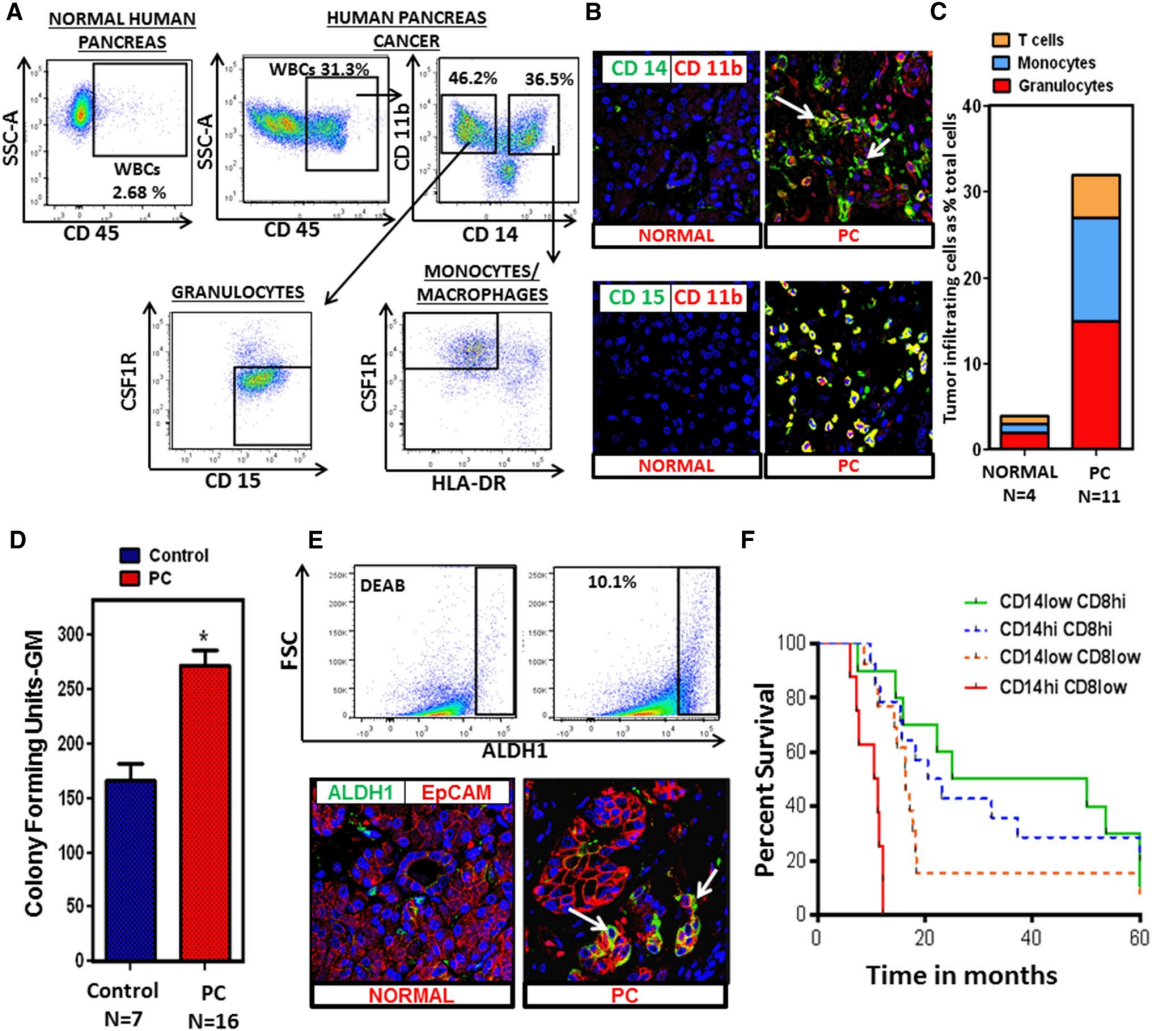
Myeloid cells and ALDH1^Bright^ cells are prevalent in PC. **a** Flow cytometry performed on human PC samples (*N* = 11) and normal human pancreas (*N* = 4). Representative flow cytometry plot of normal human pancreas and PC tumor compares CD45^+^ infiltrate. Flow cytometry plots gating on CD45^+^ cells shows CD14^+^ and CD15^+^ infiltrate in the tumor. A significant population of CD14^+^ cells is HLA-DR^low/−^. A representative flow plot shows 2.68 % CD45^+^ cells infiltrating the normal human pancreas (gating is performed using isotype control). **b** Immunofluorescent staining was performed on normal human pancreas samples (*N* = 5) and PC samples (*N* = 5). Representative immunofluorescent confocal images (×63) of normal human pancreas and PC stained for (CD11b, CD14), (CD11b, CD15). *Green* CD14 or CD15, *red* CD11b and *blue Topro*. **c** A histogram reporting percentages (average values) of the monocytic and granulocytic myeloid cells and T cells infiltrating the tumor as a percentage of total cells in PC tissue (*N* = 11) compared to normal human pancreas (*N* = 4). Granulocytes (normal pancreas (mean ± SD) = 1.87 ± 0.5 and PC = 14.6 ± 0.65, *p* value <0.05) and monocytes (normal = 1.3 ± 0.7 and PC = 12.4 ± 1.07, *p* value <0.05) are significantly increased in PC. **d** Upregulation of myelopoiesis in human PC; bone marrow specimens were collected from PC patients (*N* = 16) and age-matched healthy controls (*N* = 7); methylcellulose-based colony-forming unit assays were performed as described in “Materials and methods” section and were scored for the total number of myeloid colonies (CFU-GM) after 8 days. *Bar graph* depicts mean ± SEM and *asterisk* denotes statistically significant difference between the two groups *p* < 0.05 by Mann–Whitney test. **e** ALDH1 activity in human PC (*N* = 11); representative flow cytometry plot of human PC tumor gated on live EpCAM^+^ cells using Aldefluor assay. Immunofluorescent staining was performed on PC samples (*N* = 5) and normal human pancreas samples (*N* = 5). Representative immunofluorescence confocal images (×63) of normal human pancreas and PC stained for EpCAM, ALDH1 (*green* ALDH1, *red* EpCAM and *blue Topro*). *Arrows* point toward ducts expressing ALDH1 activity. **f** The CD14–CD8 ratio predicts PC patient survival. Automated analysis of CD14^+^ and CD8^+^ IHC reveals the relationship between leukocyte density and overall survival. The Kaplan–Meier estimate of overall survival comparing CD14^Hi^/CD8^Low^, CD14^Low^/CD8^Low^, CD14^Hi^/CD8^Hi^, and CD68^Low^/CD8^High^, is shown. Patients with predominant CD14^+^/CD8^Low^ infiltrate in the tumor had a significantly reduced overall survival compared to all other groups (denoted as CD14^Low^/CD8^Low^, CD14^Hi^/CD8^Hi^, and CD14^Low^/CD8^Hi^). There is a statistically significant difference between CD14^Hi^/CD8^Low^ and CD14^Low^/CD8^Hi^, *p* value <0.001

PC tumors possess a dense stroma characterized by abundant immunosuppressive myeloid cells [24]. However, myeloid cells are heterogeneous with different phenotypes. Therefore, we sought to better characterize these cells in human PC and found that both CD14^+^ (monocytic) and CD15^+^ (granulocytic) myeloid cells were highly prevalent by both flow cytometry analysis and immunofluorescence confocal microscopy (Fig. 1a, b). A significant population of CD14^+^ cells within the tumor had low HLA-DR expression by flow cytometry, a characteristic of Mo-MDSC (Fig. 1a). Unlike PC, however, normal human pancreas has significantly fewer number of CD45^+^ cells (including CD14^+^, CD15^+^ myeloid infiltrate) compared with human PC (Fig. 1a, c; Supplementary figures 1, 2).

We hypothesized that the high prevalence of MDSC in the tumor microenvironment was a result of enhanced myelopoiesis in the bone marrow of PC patients and active recruitment to the tumor. In order to study the effect of tumors on bone marrow myelopoiesis, we measured the myeloid progenitor cells by performing granulocyte macrophage colony-forming unit assays (CFU-GM) using bone marrow aspirates of PC patients and healthy controls. Indeed, bone marrow from PC patients formed significantly more CFU-GM compared to age-matched healthy controls (Fig. 1d). This suggests that tumors enhance myelopoiesis in the bone marrow of PC patients.

As previously stated, ALDH1 defines a subpopulation of treatment-resistant cancer cells with enhanced tumor-initiating properties in PC [14, 16]. Our group previously reported that ALDH1^Bright^ murine PC cells express higher levels of CD29, CD44, and CD49f, and we functionally characterized this population of cells by both in vitro spheroid assays and in vivo tumorigenic potential in nude mice. We also demonstrated that enrichment of ALDH1^Bright^ cells promotes chemoresistance in PC [15]. Here, we performed flow cytometry and immunofluorescence staining and found that ALDH1^Bright^ CSCs [identified as CD45^−^, EpCAM^+^ and propidium iodide (PI)^−^] composed roughly 6–10 % of tumor cells (Fig. 1e) compared to normal human pancreas (Supplementary figure 3).

To understand the clinical implications of the CD14^+^ cell infiltrate, we analyzed a tissue TMA from 60 PC patients. Tumors were scored for the presence of CD14^+^ and leukocytes and stratified into four groups (CD14^Hi^/CD8^Low^, CD14^Low^/CD8^Low^, CD14^Hi^/CD8^Hi^, and CD14^Low^/CD8^Hi^). We observed that patients with predominant CD14^+^/CD8^Low^ infiltrate in the tumor had a significantly reduced overall survival compared to all other groups (denoted as CD14^Low^/CD8^Low^, CD14^Hi^/CD8^Hi^, and CD14^Low^/CD8^Hi^, *p* value <0.001) (Fig. 1f). Further analysis showed that CD14^+^ leukocytes correlated with tumor ALDH1 expression (Spearman’s *r* = 0.2, *p* = 0.02) and tumors classified as CD14^Hi^ had increased ALDH1^Bright^ cells (Supplementary figure 4). This led us to examine the link between the abundant MDSC infiltrate and the ALDH1^Bright^ CSC sub population, which is a well-established cancer stem cell population in murine PC model [15].

### GCSFR^−/−^ mice with pancreatic cancer exhibit impaired myelopoiesis

**Fig. 2:**
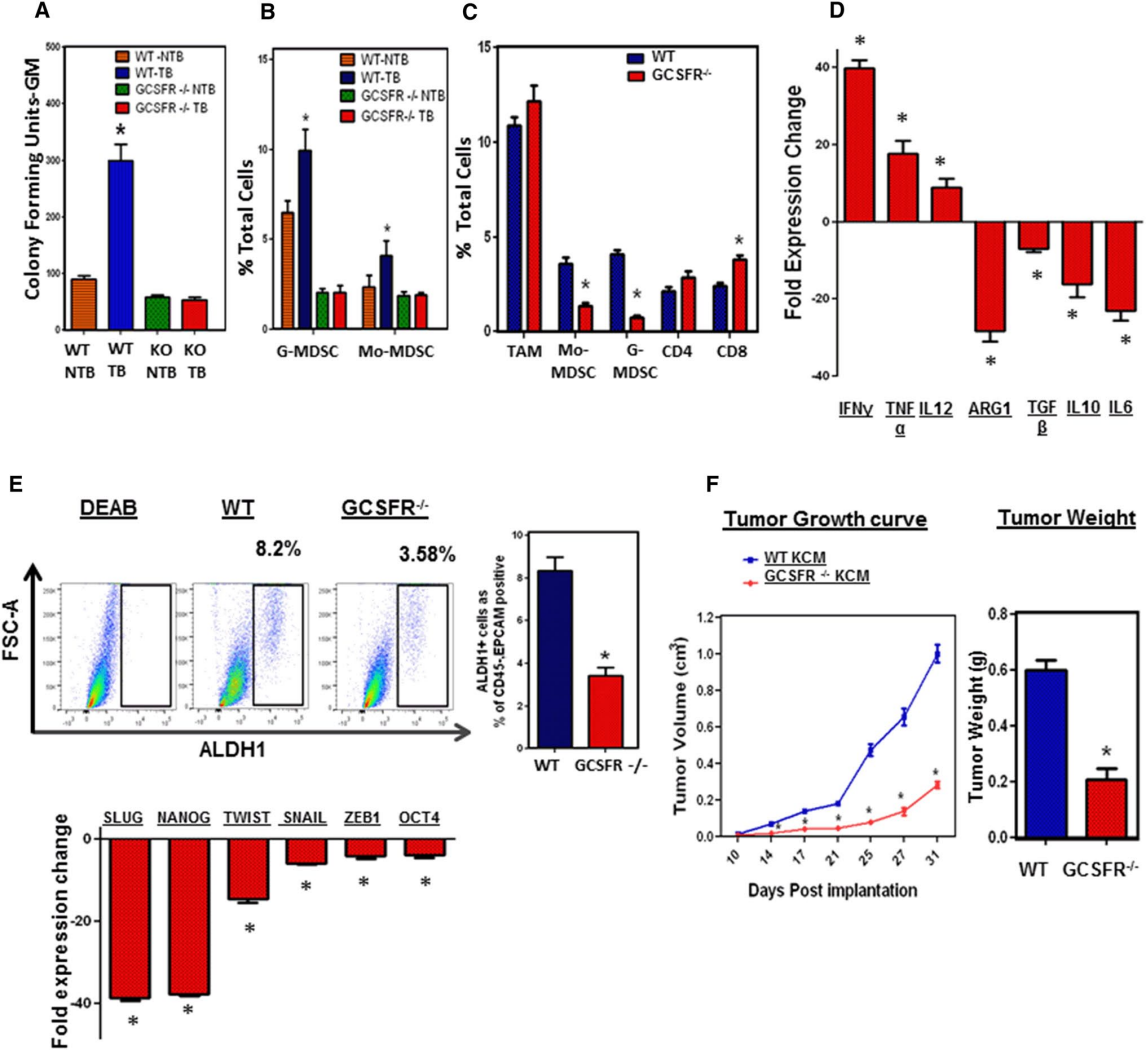
Myeloid cells promote CSCs and EMT in murine PC. **a** CFU-GM in bone marrow; bone marrow was isolated from tumor-bearing and non-tumor-bearing WT and GCSFR^−/−^ mice (*N* = 7–10 mice/group), methylcellulose-based colony-forming unit assays were performed and scored for total number of myeloid (CFU-GM) colonies after 8 days. Tumor-bearing WT mice have significantly increased myeloid colonies (CFU-GM) as compared to non-tumor-bearing WT mice. GCSFR^−/−^ mice have significantly decreased CFU-GM as compared to non-tumor-bearing WT mice (*NTB* non-tumor-bearing, *TB* tumor-bearing). **b** Peripheral blood G-MDSC (CD11b^+^/Gr1^+^/Ly6G^+^/Ly6C^mid^) and Mo-MDSC (CD11b^+^/Gr1^+^/Ly6G^−^/Ly6C^hi^) calculated as a percentage of total cells. Data are shown for NTB and TB WT and GCSFR^−/−^ mice. **c** Analysis compares tumor myeloid and lymphoid infiltrate by flow cytometry in WT and GCSFR^−/−^, KCM tumor-bearing mice. Mo-MDSC = CD11b^+^/Gr1^+^/Ly6G^−^/Ly6C^hi^/F4/80^mid^, G-MDSC = CD11b^+^/Gr1^+^/Ly6G^+^/Ly6C^mid^, T cells = CD45^+^/CD3^+^/CD4^+^, or CD8^+^, TAM = CD45^+^/CD11b^+^/F4/80^hi^/Ly6C^low^/MHCII^+^. **d** *Bar graph* shows fold gene expression change in GCSFR^−/−^ tumors relative to WT tumors. **e** Representative flow cytometry plot showing mouse orthotopic PC specimens stained for ALDH1 activity. Analysis by flow cytometry demonstrated approximately 8.2 % ALDH1^Bright^ CSCs in WT tumors and 3.58 % in GCSFR^−/−^ tumors. *Bar graph* shows fold gene expression of Slug, Nanog, Twist, Snail, ZEB-1, and OCT-4 in GCSFR^−/−^ tumors relative to WT tumors. **f** Tumor growth curves comparing subcutaneous tumor growth, KCM by caliper measurements in WT mice and GCSFR^−/−^ mice. *Points on curve* represent mean values ± SEM at indicated time points. *Bar graph* compares tumor weights of orthotopically implanted KCM in WT and GCSFR^−/−^ mice 31 days post-injection. It depicts mean ± SEM and *asterisk* denotes statistically significant difference between the two groups *p* < 0.05 by Mann–Whitney test

To further study the potential relationship between tumor-induced bone marrow myelopoiesis and increased ALDH1^Bright^ CSCs in tumors, we employed a mouse model. Granulocyte colony-stimulating factor (G-CSF/CSF-3) is a critical growth factor for myeloid cell production in the bone marrow [25, 26]. We utilized a G-CSF receptor knock out (GCSFR^−/−^) mouse model (on a C57BL/6 genetic background) to study the effects of myelopoiesis in PC. These mice are unique in that they cannot increase myelopoiesis in response to physiologic triggers, including tumors [25, 27]. We utilized three distinct murine PC cell lines, denoted as KCM, KCKO, and Pan02, derived from spontaneous PC arising from genetic models (LSL-KRAS^G12D^ × P48-Cre × MUC1 and LSL-KRAS^G12D^ × p48-Cre, respectively) or tumors arising from 3-methylcholanthrene carcinogenesis (Pan02) [23]. We implanted tumor cells subcutaneously in WT and GCSFR^−/−^ mice and isolated bone marrow from both tumor bearing and non tumor bearing mice. Tumor bearing GCSFR^−/−^ mice had significantly less CFU-GM as compared to WT mice ex vivo (Fig. 2a). Similar to PC patients, we observed an increase in bone marrow myelopoiesis and MDSC (CD11b^+^, Gr1^+^) in the peripheral blood of tumor-bearing WT mice [7]. In vivo, GCSFR^−/−^ mice were not able to upregulate myelopoiesis in response to PC implantation, which translated into significantly reduced G-MDSC and Mo-MDSC in the blood of tumor-bearing mice (Fig. 2b). However, MDSC from tumor-bearing WT and GCSFR^−/−^ mice were similarly immunosuppressive ex vivo (Supplementary figure 5). We have studied the direct ligand-receptor expression and identified that tumor cells do not express GCSF receptor (data not shown). This suggests that while GCSFR^−/−^ mice have reduced myelopoiesis, their myeloid cells retain immunosuppressive properties, thus meeting the definition of MDSC [28].

### MDSC reduction alters the tumor microenvironment and correlates with decreased frequency of ALDH1^Bright^ CSCs in murine pancreatic cancer

Because PC is characterized by an abundant stroma where myeloid cells predominate, we sought to determine the relationship between MDSC and ALDH1^Bright^ CSCs using the GCSFR^−/−^ mouse model. GCSFR^−/−^ mice have significantly decreased tumor-infiltrating G-MDSC and Mo-MDSC, compared to WT tumors. In addition, there was a corresponding increase in tumor-infiltrating CD8^+^ T cell infiltrate while CD4^+^ T cells and macrophages (defined as CD45^+^/CD11b^+^/F4/80^hi^/Ly6C^low^/MHCII^+^) were unchanged (Fig. 2c). The bar graph shows infiltrating tumor cells as a percentage of total cells. We also compared the tumor infiltrate in WT tumors that had different volumes (0.4 and 1.0 cm^3^) to rule out the possibility that the differences in the G-MDSC and Mo-MDSC infiltrate are due to different tumor sizes **(**Supplementary figure 6 D). Furthermore, GCSFR^−/−^ mice exhibit a shift in the immune profile of PC tumors from a TH-2 to TH-1 immune response, characterized by increased expression of IFN-γ, TNF-α and IL-12 with decreased expression of arginase-1, IL-6, TGF-β, IL-10 compared to WT mice (Fig. 2d). Interestingly, tumors from GCSFR^−/−^ mice had significantly reduced ALDH1^Bright^ CSCs compared to those from WT mice (Fig. 2e). GCSFR^−/−^ tumors also displayed decreased expression of genes associated with cell pluripotency (i.e. Oct-4 and Nanog) and EMT (i.e. Snail, Slug, Twist and Zeb-1 by RT-PCR) (Fig. 2e). This suggests that the absence of MDSC in GCSFR^−/−^ tumors is associated with a decrease in ALDH1^Bright^ CSCs.

Ultimately, this was associated with significantly decreased tumor growth in both subcutaneous and orthotopic PC tumors in GCSFR^−/−^ mice (Fig. 2f). These findings suggest an enhanced antitumor immune response in GCSFR^−/−^ mice is associated with reduced bone marrow-derived MDSC in the tumor. Similar results were found using other murine PC cell lines (Supplementary figure 6A–C).

### Monocytic MDSC directly increase the frequency of ALDH1^Bright^ CSCs in murine PC

**Fig. 3:**
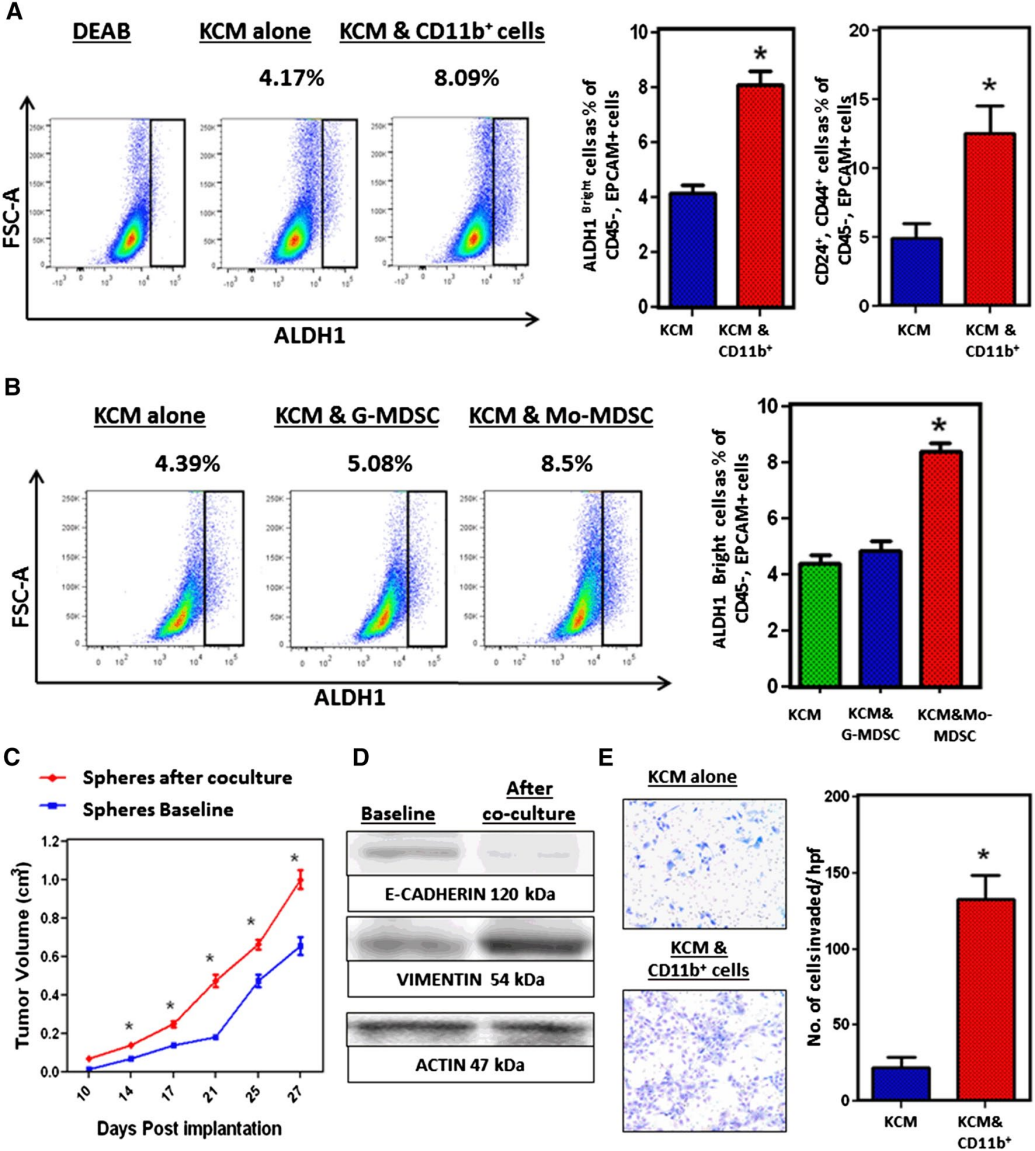
**a** KCM cultured alone and with CD11b^+^ cells isolated from bone marrow of WT tumor-bearing mice by magnetic bead isolation, for 72 h. KCM tumor cells were isolated and stained for ALDH1 activity and other CSC markers. Representative flow cytometry plots show ALDH1^Bright^ CSCs, which constitute approximately 4.17 % when KCM is cultured alone and increase up to 8.09 % after coculture with CD11b^+^ cells. *Bar graph* shows percentage ALDH1^Bright^ and CD24^+^, CD44^+^ cancer stem cells in KCM, with and without coculture with CD11b^+^ cells. **b** G- and Mo-MDSC were isolated from orthotopically implanted KCM tumors of WT mice by FACS. These cells were separately cocultured with KCM for 72 h. After 72 h, KCM cells were isolated and stained for ALDH1 activity. Representative flow cytometry plots show that ALDH1^Bright^ CSCs constitute approximately 4.39 % when KCM is cultured alone, 5.08 % when KCM cocultured with G-MDSC and increases up to 8.5 % when KCM is cocultured with Mo-MDSC. **c** Tumor growth *curves* comparing subcutaneous tumor growth, KCM spheres (with baseline ALDH1 activity) and KCM spheres after coculture (increased ALDH1 activity) by caliper measurements in WT mice. *Points on curve* represent mean values ± SEM at indicated time points. **d** CD11b^+^ cells were isolated after coculture with tumor cells in transwell plates and were used for Western blotting. Western blot analysis of KCM shows downregulation of E-cadherin and upregulation of vimentin in KCM from baseline after coculture with CD11b^+^ cells. **e** Invasion assays were performed to compare the invading properties of tumor cells. Tumor cells were seeded in the upper part of Matrigel-coated transwell inserts. Cell invasion index was calculated as the number of cells attached to the *bottom* of Matrigel-coated membrane. Figure shows that KCM tumor cells have increased invasion through Matrigel matrix membrane after coculture relative to tumor cells alone. Cells per high-power field were quantified. Cells were counted in six different fields of triplicate membranes. *Graph* depicts the number of invaded cells per high-powered field (mean ± SEM of three independent experiments). *Asterisks* denotes statistically significant difference between the two groups *p* < 0.05 by Mann–Whitney test. We used 7–10 mice per group and all these experiments were repeated in triplicate

Since decreased tumor-infiltrating MDSC were associated with reduced ALDH1^Bright^ CSCs, we hypothesized that MDSC contribute to expanding the population of ALDH1^Bright^ CSCs. To test this, we utilized an in vitro model utilizing murine cell lines in order to exclude the possibility of involvement of other cell types. We cocultured in vitro-maintained KCM with myeloid cells from the bone marrow of tumor-bearing mice (cells were separated by transwell insert). The frequency of ALDH1^Bright^ CSCs and CD24^+^, CD44^+^ CSCs was significantly increased in KCM cocultured with CD11b^+^ cells, compared to KCM alone (Fig. 3a). However, MDSC are composed of both Mo-MDSC and G-MDSC and perhaps only one of these two subsets were responsible for this observation. Therefore, we isolated Mo-MDSC and G-MDSC from the tumors of WT mice by FACS and cocultured these cells separately with KCM. We found that the frequency of ALDH1^Bright^ CSCs was significantly increased when KCM was cocultured with Mo-MDSC while there was no increase with G-MDSC, separated by transwell insert (Fig. 3b). This suggests that Mo-MDSC enhance the ALDH1^Bright^ and CD24^+^, CD44^+^ CSCs in PC. In order to validate this finding, we also cultured murine PC tumor cells for sphere formation after they were cocultured with myeloids. The spheres were harvested and then implanted subcutaneously in WT mice, and tumor growth was compared to non cocultured tumor cells. The tumors grew faster when the tumor spheres were implanted after coculture with myeloid cells compared to the spheres that were not cocultured with myeloid cells (Fig. 3c). We also assessed changes in the expression of markers promoting epithelial to mesenchymal transition and performed functional studies. The tumor cells were separated from Mo-MDSC after coculture, and RT-PCR and Western blotting were performed. There was downregulation of E-cadherin and upregulation of vimentin in the tumor cells that were previously cocultured (separated by a transwell insert) with myeloid cells (Fig. 3d). Invasion studies further supported that tumor cells that were previously cocultured with myeloid cells had a mesenchymal phenotype and significantly invaded the Matrigel plug compared to the tumor cells that were not cocultured with myeloid cells (Fig. 3e). This suggests that Mo-MDSC promote CSCs in murine PC and promote mesenchymal properties of tumor cells.

### Human pancreatic cancer converts circulating monocytes into Mo-MDSC

**Fig. 4:**
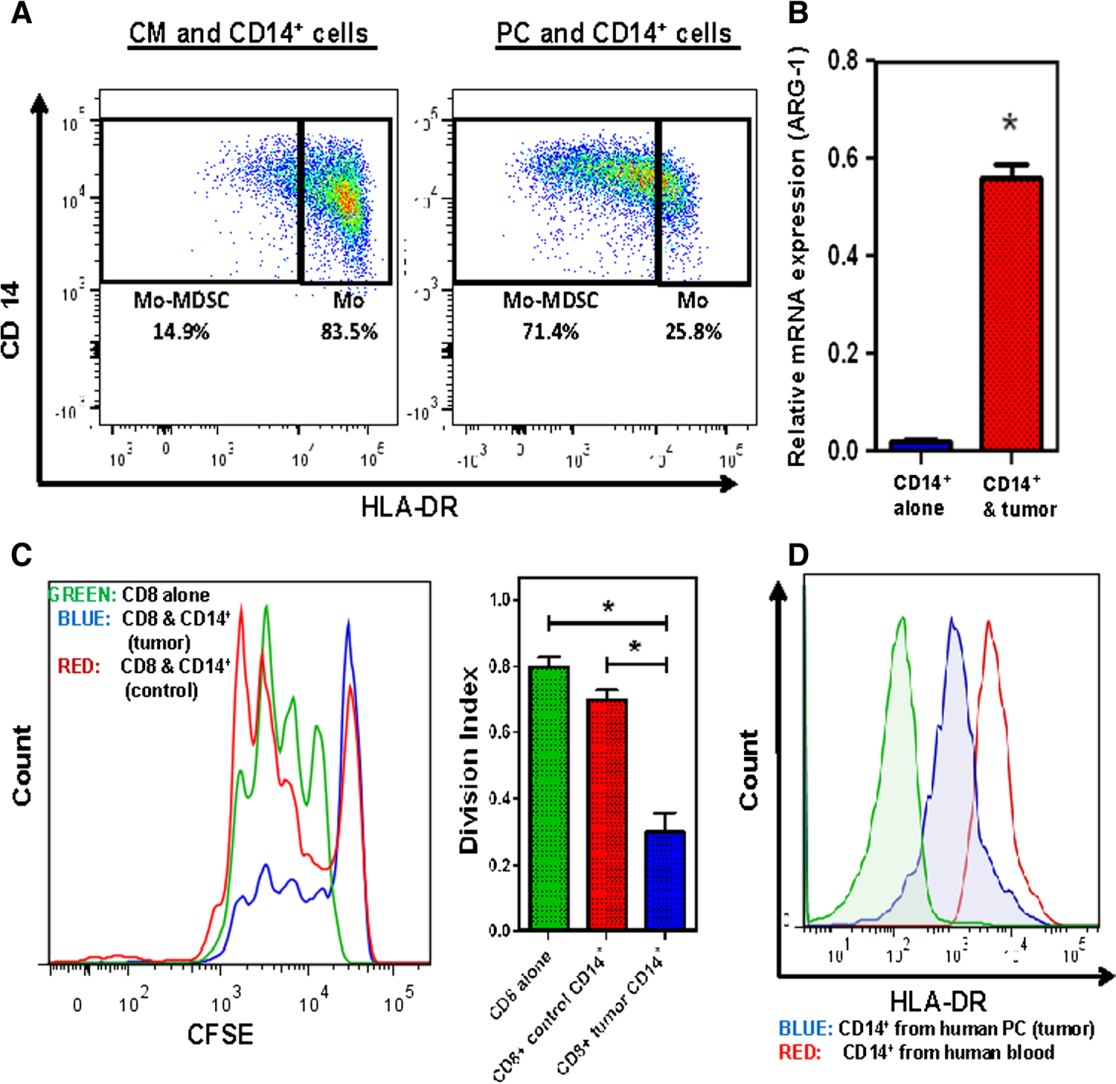
CD14^+^ cells were isolated from normal human PBMC by magnetic bead isolation and were cocultured with human PC cell line Panc-1 for 72 h. **a** Downregulation of HLA-DR expression after tumor exposure; flow cytometry was performed on CD14^+^ cells (from blood) after 72 h of coculture with tumor cells. Representative plots show that approximately 83.5 % CD14^+^ cells had high HLA-DR expression when these cells are incubated in complete medium (CM). After tumor exposure, these cells downregulate HLA-DR expression and 71.4 % CD14^+^ cells have low HLA-DR expression. **b** Analysis of quantitative RT-PCR results for gene expression of arginase-1 from CD14^+^ cells cocultured with and without tumor. Normalized fold change is depicted. **c** Representative flow cytometry histogram from T cell suppression assay depicts stimulated, CFSE-labeled CD8^+^ T cells cocultured with tumor-conditioned or unconditioned CD14^+^ cells for 72 h. **d** Representative flow cytometry plot shows differential expression of HLA-DR on CD14^+^ cells isolated from the blood and human tumor specimens. *Bar graphs* depict mean division index ± SEM with *asterisk* denotes *p* < 0.05 by Mann–Whitney in all *panels*

In order to study the role of Mo-MDSC in human PC, we cocultured normal human blood monocytes with human PC cell lines (Panc-1 and BxPC3). The CD14^+^ cells were harvested and assessed by flow cytometry, immune suppression, RT-PCR, and Western blotting. These monocytes acquired a Mo-MDSC phenotype characterized by a significantly decreased expression of HLA-DR and increased expression of arginase-1 as well as the ability to suppress CD8^+^ T cell proliferation in vitro [29–32] (Fig. 4a–c). See supplementary figure 7 for similar results using the BxPC3 cell line. Thus, tumor-derived factors in PC are capable of changing the phenotype and functionality of human blood monocytes, converting these cells into immunosuppressive Mo-MDSC. In PC patients with primary disease only, the CD14^+^ cells in the blood have a high HLA-DR expression but these cells only obtain MDSC phenotypic markers when they infiltrate the tumor (Fig. 4d).

### Mo-MDSC promote stemness in human PC by a STAT3-dependent mechanism

**Fig. 5:**
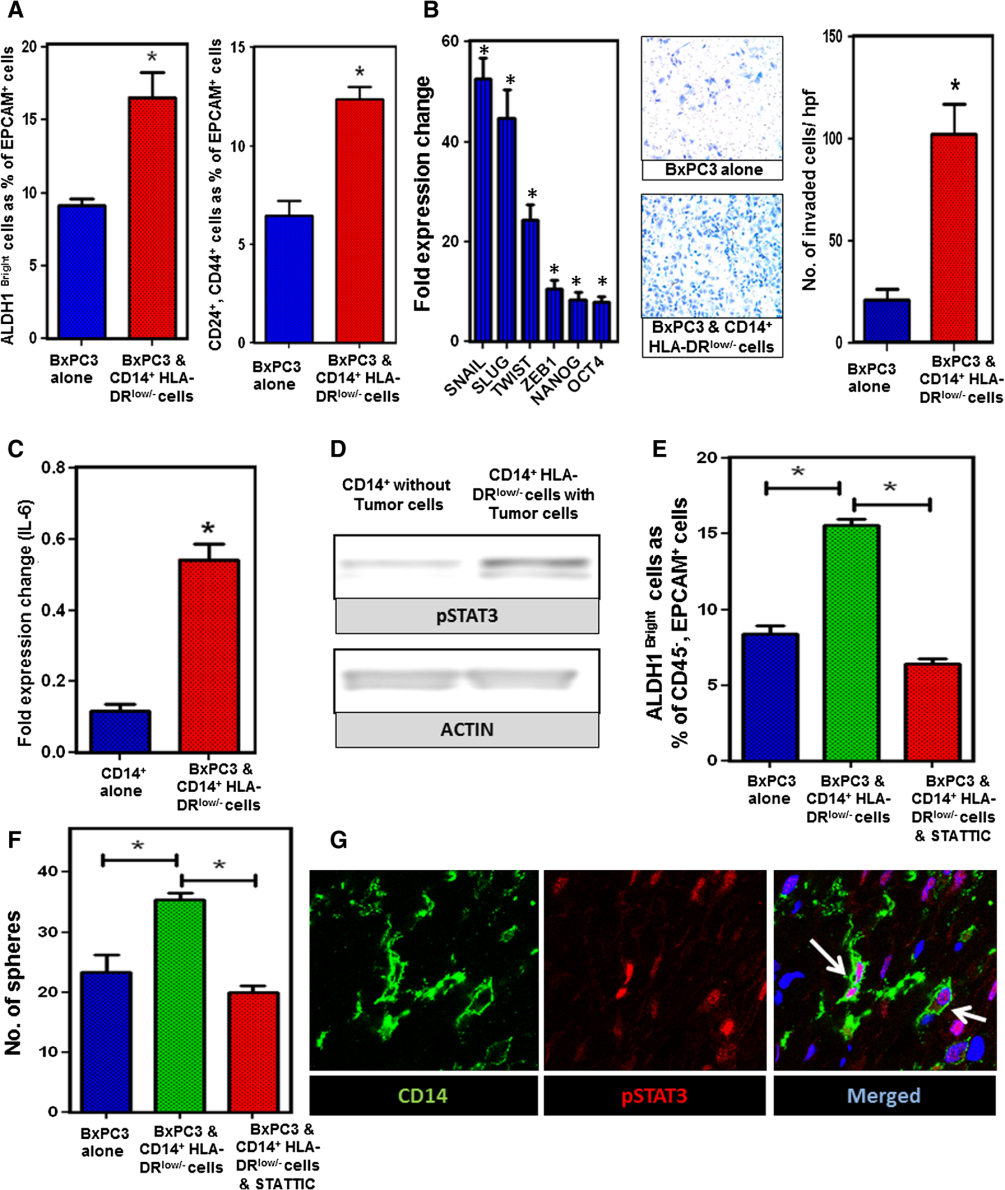
STAT3 activity in Mo-MDSC promotes CSCs and EMT in PC. a CD14^+^/HLA-DR^low/−^ cells were cocultured with BxPC3 for 72 h and ALDH1, CD24, and CD44 staining was performed. Graph shows ALDH1^Bright^ CSCs and CD24^+^, CD44^+^ cells as a percentage of CD45^−^, EpCAM^+^, PI^−^ cells. **b** Tumor EMT markers and invasion assay; RT-PCR shows that markers of cell pluripotency and EMT were upregulated in BxPC3 tumor cells after coculture with CD14^+^/HLA-DR^low/−^ cells. Invasion assays showing that BxPC3 tumor cells have increased invasion through Matrigel matrix membrane after coculture with CD14^+^ cells relative to tumor cells alone. Cells per high-power field were quantified. *Graph* depicts the number of invaded cells per high-powered field (mean ± SEM of three independent experiments). **c** Analysis of quantitative RT-PCR results for expression of IL-6 by CD14^+^ cells cocultured with and without BxPC3 tumor. Normalized fold change is depicted. **d** Western blot analysis of STAT3 phosphorylation in CD14^+^ cells cocultured with and without BxPC3 tumor cells for 72 h. **e** Inhibition of STAT3 signaling in CD14^+^/HLA-DR^low/−^ cells prevents the increase in ALDH1^Bright^ CSCs in BxPC3 from baseline. CD14^+^ cells treated with STATTIC were washed and then cocultured with tumor cells in transwell plates. *Bar graph* shows ALDH1^Bright^ CSC population (gated on CD45^−^, EpCAM^+^, PI^−^ cells) which is approximately 8.79 % in BxPC3 alone, 15.8 % when BxPC3 was cocultured with CD14^+^/HLA-DR^low/−^ cells and 6.02 % when BxPC3 was cocultured with STATTIC-treated CD14^+^/HLA-DR^low/−^ cells. *Graph* shows that inhibition of STAT3 signaling by STATTIC (20 μM) blocks the increase in frequency of ALDH1^Bright^ cells from baseline. **f** *Bar graph* shows tumor spheroid formation in BxPC3 and Panc-1 cells with and without CD14^+^/HLA-DR^low/−^ cells in the coculture. The mean number of tumor spheroids formatted after 10 days is depicted. **g** Representative immunofluorescence confocal images (×63) of human PC stained for CD14^+^ cells and pSTAT3 activity. *Green* CD14 and *red* pSTAT3. *Bar graphs* depict mean division index ± SEM with *asterisk* denotes *p* < 0.05 by Mann–Whitney in all *panels*

Since Mo-MDSC in mice increase the prevalence of ALDH1^Bright^ CSCs and enhance expression of genes associated with EMT in murine PC, we sought to determine if the same phenomenon occurs in human PC. We generated Mo-MDSC in vitro as described above and cocultured these cells with PC cell lines in vitro. Both Mo-MDSC and tumor cells were analyzed by flow cytometry and RT-PCR after coculture. Consistent with our findings in mice, Mo-MDSC increased the ALDH1^Bright^ and CD24^+^, CD44^+^ CSC population in BxPC3 after coculture as compared to control tumor cells (Fig. 5a). In order to validate this finding, we also cultured human PC tumor cells for sphere formation after they were cocultured with CD14^+^ cells. The spheres were isolated and implanted subcutaneously in NU/J mice and tumor growth was compared to non-cocultured tumor cells. The tumors grew faster when the tumor spheres were implanted after coculture with Mo-MDSC, compared to spheres that were not cocultured with Mo-MDSC (Supplementary figure 8). Consistent with enhanced tumor-initiating properties, we observed that the transcription factors associated with cell pluripotency and EMT, i.e., Snail, Slug, Twist, Zeb-1, Nanog, and Oct-4 were significantly increased by RT-PCR in PC cells cocultured with Mo-MDSC. Invasion studies showed that the tumor cells that were previously cocultured with Mo-MDSC have significantly increased invasion activity (Fig. 5b). Similar results were seen using Panc-1 (Supplementary figure 9). This suggests that human Mo-MDSC promote tumor stemness and EMT. Additionally, we found that IL-6 expression was upregulated in the Mo-MDSC as compared to normal monocytes (Fig. 5c).

STAT3 activity in tumor cells has been demonstrated to facilitate the expansion and maintenance of CSCs in several tumor types [17]. However, we hypothesized that up regulation of signal transducer and activator of transcription 3 (pSTAT3) in Mo-MDSC is involved in promoting ALDH1^Bright^ CSCs in PC. CD14^+^ cells were isolated from tumor cells after coculture and Western blotting was performed. We observed that there was upregulation of phosphorylated STAT3 in tumor-treated CD14^+^/HLA-DR^low/−^ cells as compared to untreated CD14^+^ cells by Western blotting (Fig. 5d). We also found upregulation of pSTAT3 in CD11b^+^ cells after coculture with murine PC cell lines (Supplementary figure 10). In order to determine if pSTAT3 mediated the ability of Mo-MDSC to increase the prevalence of ALDH1^Bright^ CSCs, Mo-MDSC were treated with STATTIC, a small molecule inhibitor of STAT3 [21] and washed with PBS. STATTIC-treated cells were then plated in a 6-well plate, and a transwell insert was placed on top of the cells. Human PC tumor cells were added to the transwell insert and were cultured for 72 h. We found that treatment with STATTIC entirely prevented the Mo-MDSC mediated increase in ALDH1^Bright^ CSCs (Fig. 5e). In addition, we performed tumor sphere-forming assays, which display an increased tumor spheroid formation compared to control tumor cells in vitro, and treatment with STATTIC significantly decreased the Mo-MDSC mediated increase in sphere formation (Fig. 5f).

STAT3 signaling in MDSC can be modulated by IL-6 [33, 34], which has been shown to enhance CSCs and EMT in cancer. While we found that IL-6 mRNA gene expression was significantly downregulated by STAT3 inhibition (Supplementary figure 11), IL-6 blockade using anti-IL-6 antibodies decreased but did not fully reverse the effect of Mo-MDSC on increasing the prevalence of ALDH1^Bright^ CSCs. This suggests that the effects of Mo-MDSC on promoting tumor stemness are likely mediated by multiple factors downstream of pSTAT3. Finally, to confirm that CD14^+^ cells express pSTAT3 in vivo, we performed immunofluorescence staining for CD14 and pSTAT3 in human PC. Indeed, the majority of CD14^+^ leukocytes in the tumor microenvironment expressed pSTAT3 (Fig. 5g). These data suggest that pSTAT3 positive Mo-MDSC play an important role in PC progression by enhancing the population of ALDH1^Bright^ CSCs and promoting EMT.

## Discussion

PC is unique for its dense infiltrate of myeloid cells with a paucity of T cells. Myeloid recruitment is critical to the establishment and progression of PC. Tumors communicate with the bone marrow to increase myelopoiesis in both tumor-bearing mice and human PC patients. These myeloid cells are then recruited to the tumor microenvironment where they enhance growth and metastasis. In this study, we demonstrate the importance of MDSC on promoting the ALDH1^Bright^ CSCs in PC tumors. In our GCSFR^−/−^ mouse model, we found that the reduction in MDSC in the tumor decreases the ALDH1^Bright^ CSC population. We also found that the expression of EMT markers and stem cell genes were downregulated in the tumors of GCSFR^−/−^ mice. We further demonstrate that Mo-MDSC in human PC promote cancer stemness in a STAT3-dependent manner.

**Fig. 6:**
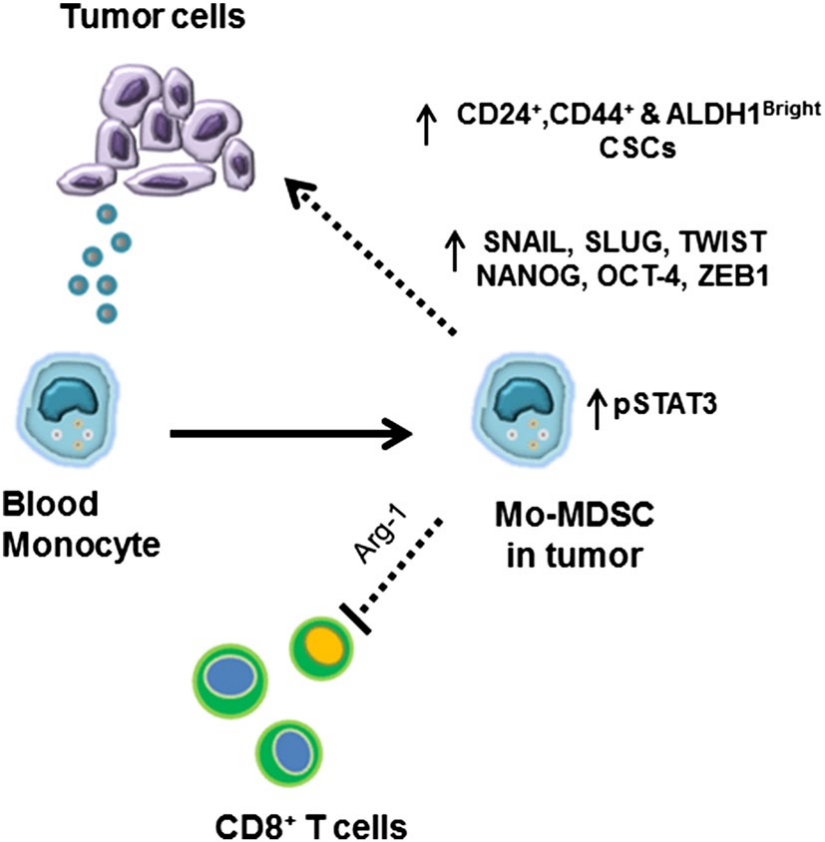
Activation of pSTAT3 in the Mo-MDSC enhances the CSCs in the PC tumor

In our experiments, we found that monocytes can become Mo-MDSC, as they downregulate HLA-DR expression and suppress T cell proliferation in the presence of tumor cells or tumor-derived factors (Fig. 6). This is important as it suggests that even mature human monocytes can acquire immunosuppressive properties upon exposure to tumor-derived factors. We have previously demonstrated that increased peripheral blood monocyte counts correlate with decreased survival in PC patients [35]. The data presented here suggest that the phenotype and functional traits of human monocytes depend on the immune environment to which these cells are exposed. Therefore, targeting monocytes in the circulation is an attractive and novel approach, which could decrease Mo-MDSC in the tumor microenvironment.

ALDH1 enzyme activity defines a subpopulation of tumor cells which exhibit stem cell-like properties and whose prevalence correlates with survival in PC Additionally, a number of cell surface markers are co-expressed with ALDH1, such as CD44, CD24, CD133, and CXCR4 [36, 37]. Evidence in mice suggests that ALDH1 has an important role in the embryological development of the pancreas [38, 39]. However, a subpopulation of ALDH1^Bright^ CSCs exhibit enhanced chemoresistance and metastasis [15]. Despite much progress, further work is needed to find better phenotypic and functional markers of CSCs in human malignancy.

STAT3 is a crucial transcription factor involved in inflammation. In murine models, pSTAT3 has been shown to regulate the expansion of myeloid progenitors as well as MDSC [40, 41].

Mo-MDSC depend on STAT3 for their immunosuppressive properties, such as the production of arginase-1 [30]. We show here that STAT3 activation in Mo-MDSC is also critical for enhancing the CSC population in PC. It is likely that STAT3 phosphorylation reprograms monocytes to acquire a pro-tumor, immunosuppressive (MDSC) phenotype. We show that STAT3 inhibition completely abrogates the ability of Mo-MDSC to enhance the ALDH1^Bright^ CSC population in vitro.

Bone marrow-derived myeloid cells make up a significant proportion of the stroma in solid tumors where they promote proliferation and survival of tumor cells. Recent reports demonstrate that tumor-infiltrating macrophages support early tumorigenesis by inducing EMT through TGF-β signaling [42]. In addition, other studies have shown that there is cross talk between MDSC and macrophages that polarizes macrophages into an M2 phenotype [43] which contribute to EMT [44]. Like macrophages, the high frequency of Mo-MDSC-infiltrating tumors suggests these cells are critical in mediating immune suppression in cancer patients. Our murine model and in vitro studies confirm that in addition to the direct suppression of T cells, Mo-MDSC also play an important role in promoting tumor stemness and EMT in a STAT3-dependent manner and this effect is independent of any other cell type in the tumor. Our data further show that Mo-MDSC and macrophages can be distinguished based on expression of HLA-DR (Figs. 4, 5). Thus, we conclude that both Mo-MDSC and macrophages promote EMT, but further studies are required to assess possible mechanistic, spatial, and/or temporal differences.

Overall, our study shows that Mo-MDSC in both mice and humans promote ALDH1^Bright^ CSCs in PC through STAT3 activation. Therefore, an improved understanding of the interactions between MDSC and CSCs is crucial for the development of novel therapies that may intercept this communication. Our findings suggest that there are multiple pathophysiologic pathways, which could be therapeutically targeted in PC. Blocking tumor-induced myelopoiesis [45], the recruitment of monocytes to the tumor microenvironment, e.g., through blockade of chemokine receptors, the conversion of monocytes into Mo-MDSC, and the STAT3-dependent enhancement of tumor stemness all offer opportunities to thwart the tumor-promoting effects on myeloid cells in this disease. In conclusion, we show that monocytes can become Mo-MDSC in the presence of tumor by acquiring immunosuppressive properties. Furthermore, Mo-MDSC infiltrate tumors where they promote cancer stemness in a STAT3-dependent manner.

## Electronic supplementary material

Below is the link to the electronic supplementary material.

Supplementary material 1 (PDF 807 kb)

## Abbreviations

CCR2: Chemokine (C–C motif) receptor 2
CSC: Cancer stem cell
G-CSF: Granulocyte colony-stimulating factor (CSF-3)
G-MDSC: Granulocytic myeloid-derived suppressor cell
Mo-MDSC: Monocytic myeloid-derived suppressor cell
PC: Pancreatic cancer
STAT3: Signal transducer and activator of transcription 3
TAM: Tumor-associated macrophage
