# Supplementary material for: Tumor-induced STAT3 activation in monocytic myeloid-derived suppressor cells enhances stemness and mesenchymal properties in human pancreatic cancer
Source: Cancer Immunol Immunother. 2014 Mar 21;63(5):513–28. doi: 10.1007/s00262-014-1527-x (PMC3994288; doi:10.1007/s00262-014-1527-x)
Supplement: Supplementary file 1 — Supplementary material 1 (PDF 807 kb) [file 262_2014_1527_MOESM1_ESM.pdf]

Panni et. al.
Supplementary figure: 1

Normal Human Pancreas

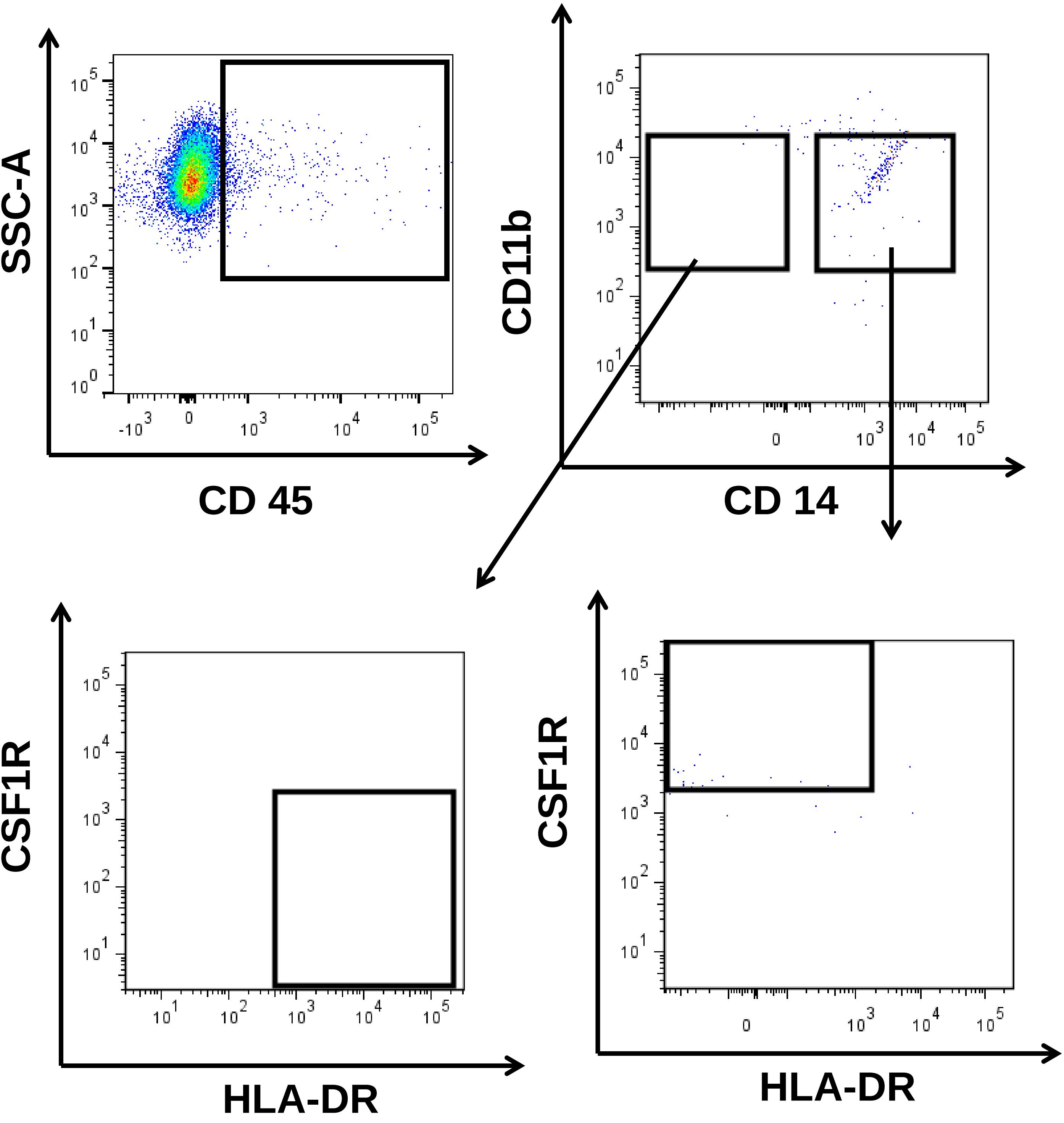

Supplementary figure 1: Flow cytometry performed on normal human pancreas samples (N=4). A representative flow plot shows 2.89% $CD45^+$ cells infiltrating the normal human pancreas (gating is performed using isotype control).

Panni et. al.
Supplementary figure: 2

NORMAL HUMAN PANCREAS (40X)
BROWN=CD 14

Normal # 1 | Normal # 2 | Normal # 3 | Normal # 4 | Normal # 5

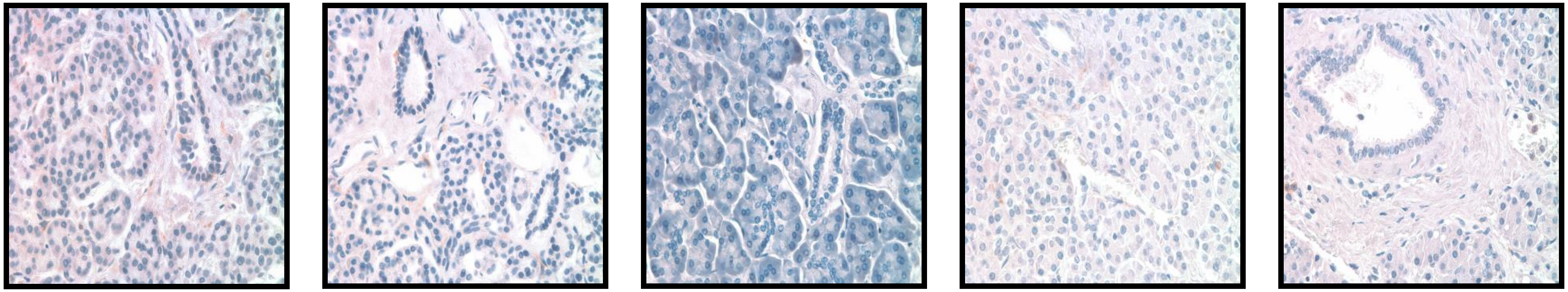

HUMAN PANCREATIC CANCER

PC pt # 1 | PC pt # 2 | PC pt # 3 | PC pt # 4 | PC pt # 5

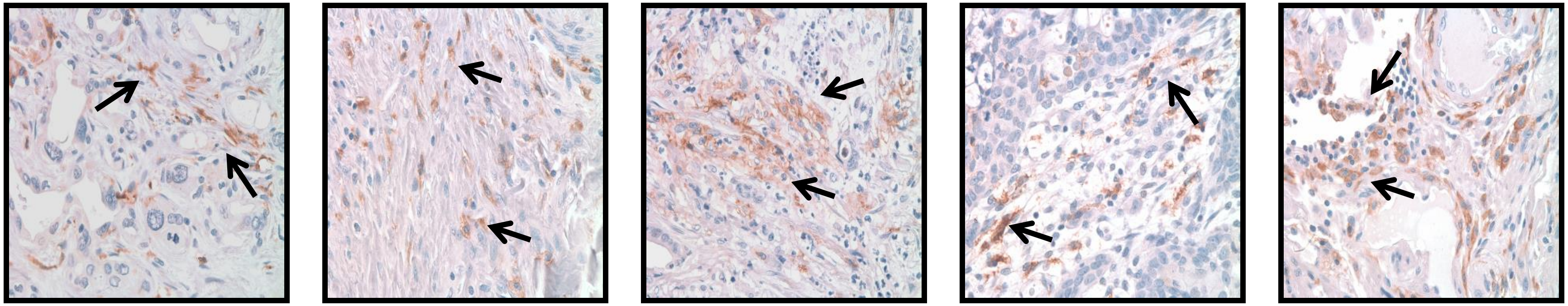

Supplementary figure 2: Immunohistochemistry images (40X) of PC tumor specimens (N=5) and normal human pancreas (N=5) stained for $CD14^{+}$ cells (Brown). Arrows point towards $CD14^{+}$ cells in the tumor.

Panni et. al.
Supplementary figure: 3

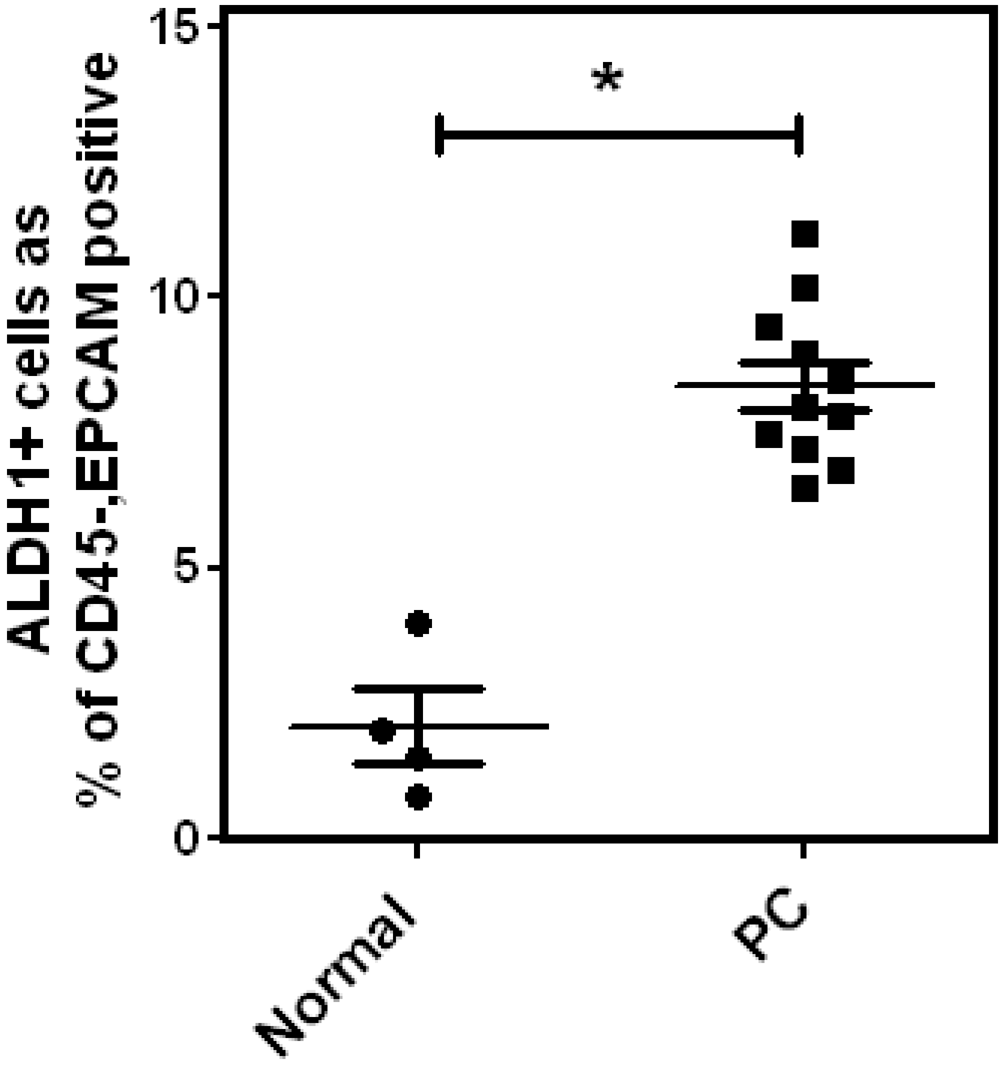

Supplementary figure 3:

A scatter plot comparing ALDH1^{Bright} cells as percent of EpCAM positive cells in PC and normal human pancreas by flow cytometry. The ALDH1^{Bright} population (as percent of EpCAM positive cells) is significantly higher in PC compared to Normal human pancreas ($p=<0.05$ by Mann Whitney test).

Panni et. al.
Supplementary figure: 4

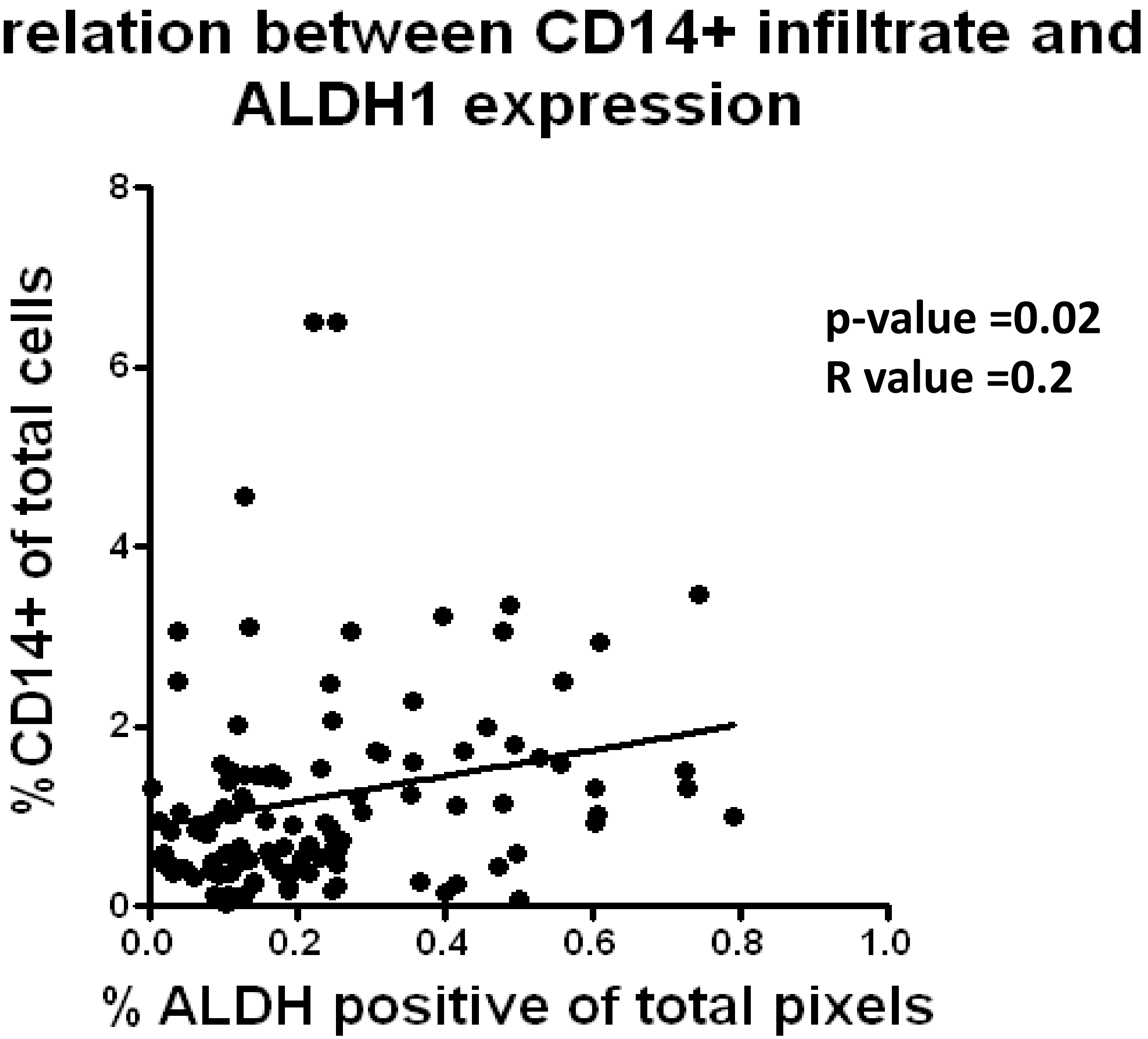

Supplementary Figure 4: Pearson correlation analysis shows a significant positive correlation of percent $CD14^+$ cells versus $ALDH1^{Bright}$ cells (Spearman r=0.2, p=0.02).

Panni et. al.
Supplementary figure: 5

RED: Splenocytes alone
BLUE: Splenocytes & TCM (GCSFR$^{-/-}$)CD11b^{+}
ORANGE: Splenocytes & TCM (WT)CD11b^{+}

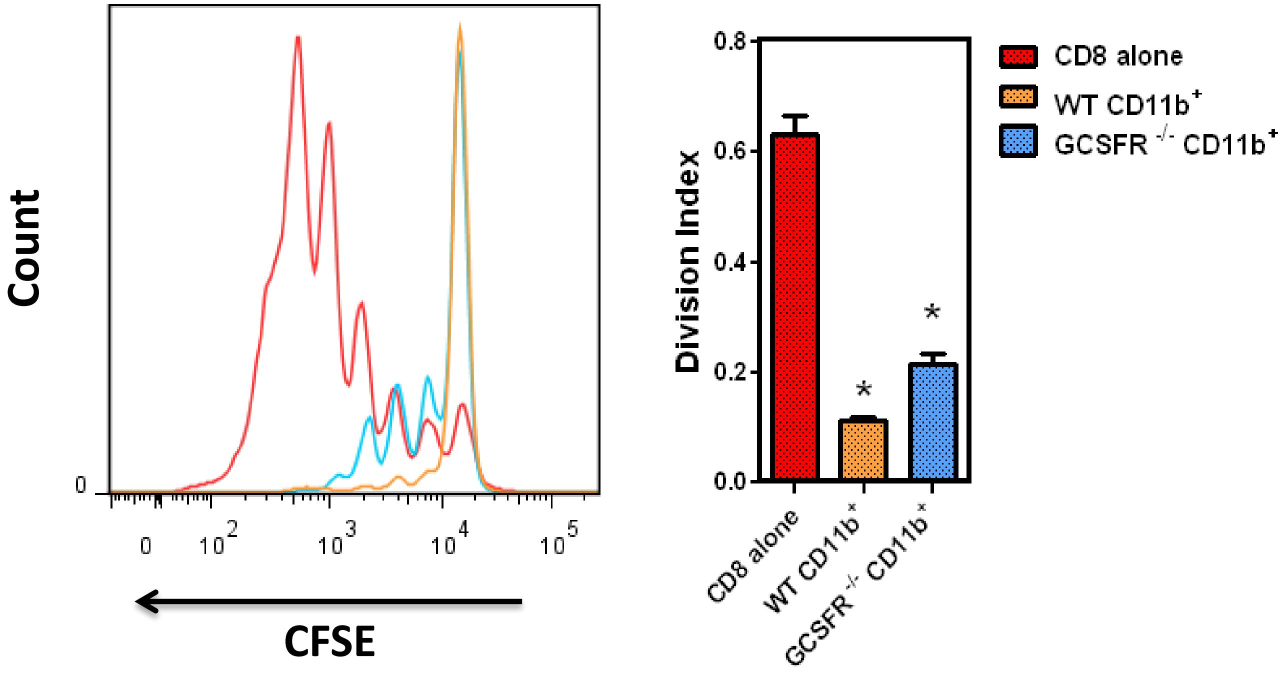

Supplementary Figure 5:

Representative CFSE dilution FACS analysis of splenocytes stimulated with a-CD3 and cultured alone or in the presence of tumor conditioned medium (TCM) and $CD11b^{+}$ cells from either $GCSFR^{-/-}$ or WT mice.

Graphs depict means ± SEM with asterisk (*) denoting statistically significant differences between groups defined as $p<0.05$ by Mann-Whitney test.

Panni et. al.
Supplementary Figure 6

A. TUMOR GROWTH CURVE (Pan02)

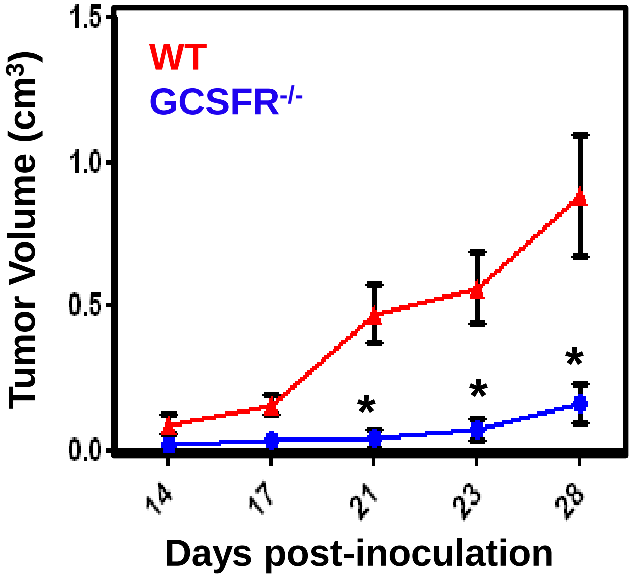

B. TUMOR WEIGHT (Pan02)

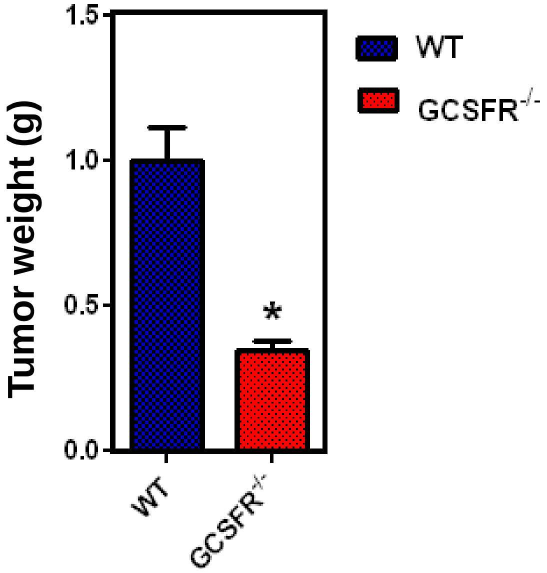

C. TUMOR ASSOCIATED MYELOID AND LYMPHOID INFILTRATE (Pan02)

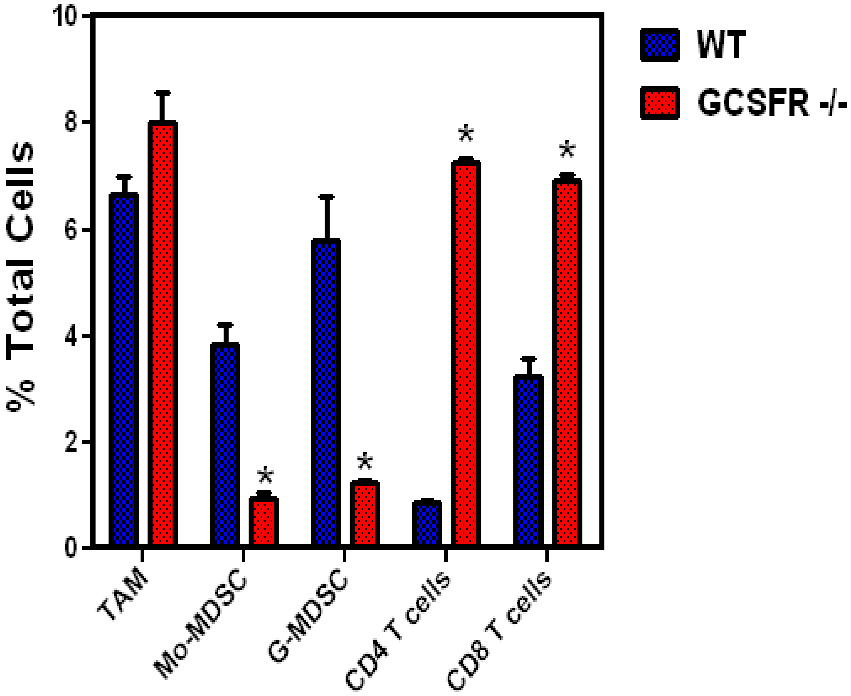

D. MYELOID INFILTRATE IN KCM TUMORS

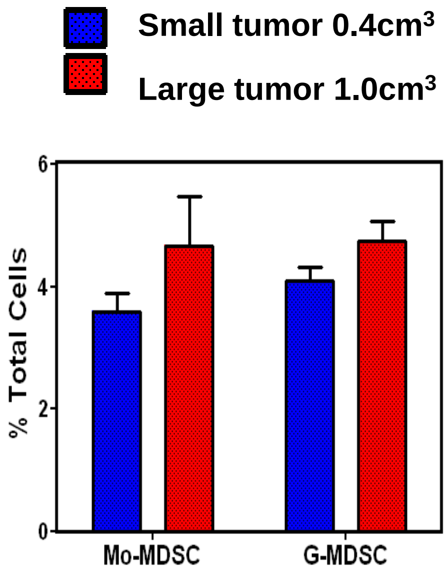

Supplementary Figure 6:

A. Tumor growth curves comparing subcutaneous tumor growth, Pan02 by caliper measurements in WT mice and GCSFR$^{-/-}$ mice. Points on curve represent mean values± SEM at indicated time points.

B. Bar graph compares tumor weights of orthotopically implanted Pan02 in WT and GCSFR$^{-/-}$ mice 28 days post injection.

C. Analysis compares tumor myeloid and lymphoid infiltrate by flow cytometry in WT and GCSFR$^{-/-}$, Pan02 tumor-bearing mice. Mo-MDSC= CD11b^{+}/Ly6C^{hi}/ Gr1^{+}/ F4/80^{mid}, G-MDSC = CD11b^{+}/Gr-1^{+}/Ly6G^{hi}/Ly6C^{mid}, T cells= CD45^{+}/CD3^{+}; TAM= CD45^{+}/CD11b^{+}/F4/80^{hi}/Ly6C^{low}/MHCII^{+}.

D. Analysis compares tumor myeloid infiltrate by flow cytometry in WT tumors. Blue bars show G-MDSC and Mo-MDSC in a small sized KCM tumor (volume= 0.4cm^{3}) and red bars show myeloid cells in large tumor (volume= 1.0cm^{3}).

Graphs depict means ± SEM with asterisk (*) denoting statistically significant differences between groups defined as $p<0.05$ by Mann-Whitney test.

Panni et. al.
Supplementary figure: 7

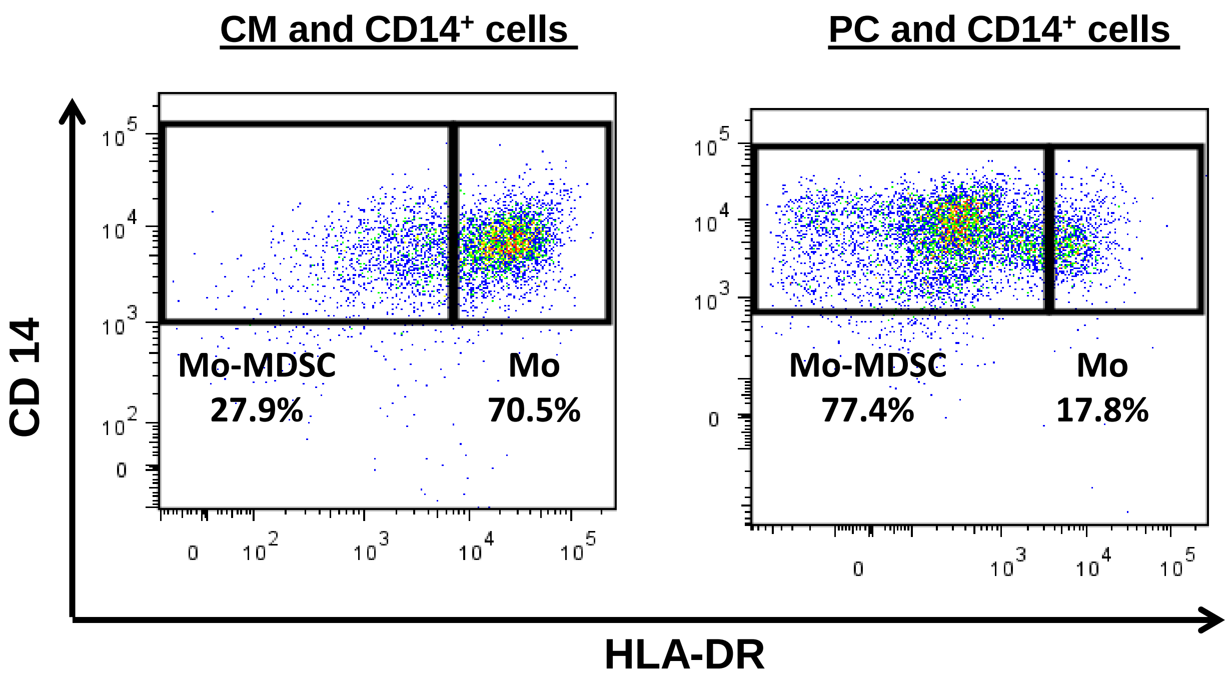

Supplementary figure 7: $CD14^+$ cells were isolated from normal human PBMC by magnetic bead isolation and were co-cultured with human PC cell line BxPC3 for 72 hours. Downregulation of HLA-DR expression after tumor exposure; flow-cytometry was performed on $CD14^+$ cells (from blood) after 72 hours of co-culture with tumor cells. Representative plots show that approximately 70.5% $CD14^+$ cells had high HLA-DR expression when these cells are incubated in complete medium (CM). After tumor exposure, these cells downregulate HLA-DR expression and 77.4% $CD14^+$ cells have low HLA-DR expression.

Panni et. al.
Supplementary figure: 8

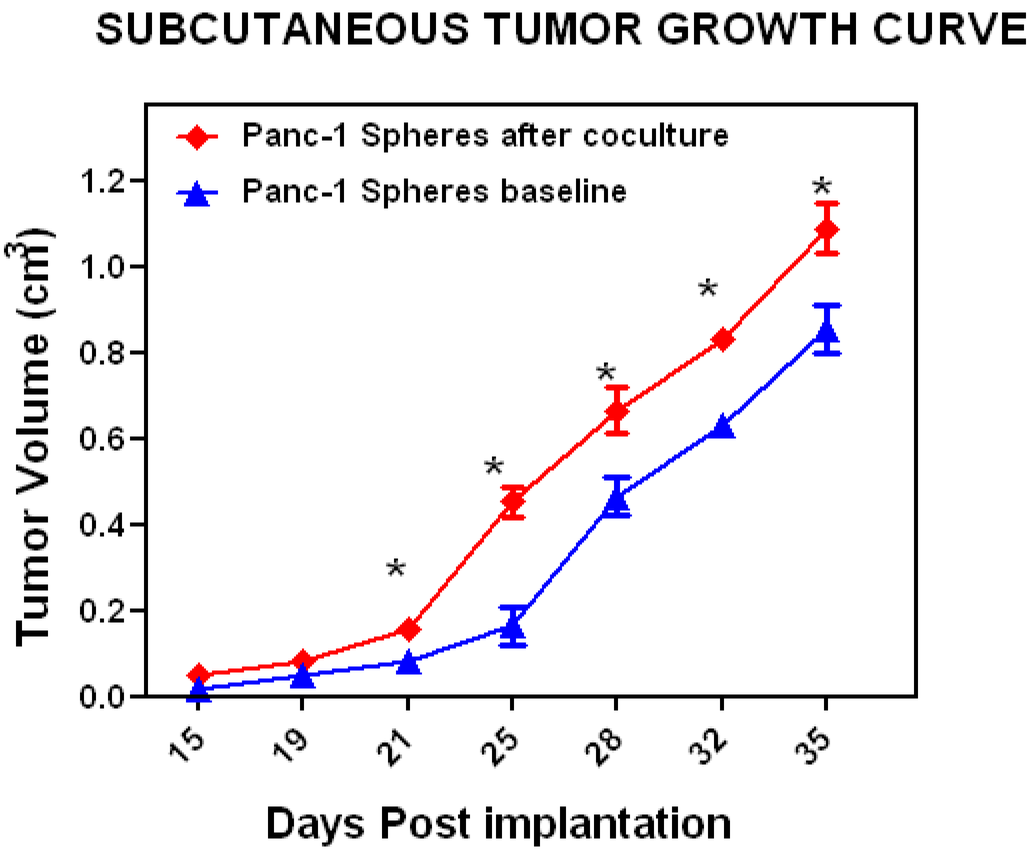

Supplementary Figure 8: Tumor growth curves comparing subcutaneous tumor growth, Panc-1 spheres (with baseline ALDH1 activity) and Panc-1 spheres after co-culture (increased ALDH1 activity) by caliper measurements in NU/J mice. Points on curve represent mean values ± SEM are indicated time points.

Panni et. al.
Supplementary Figure 9

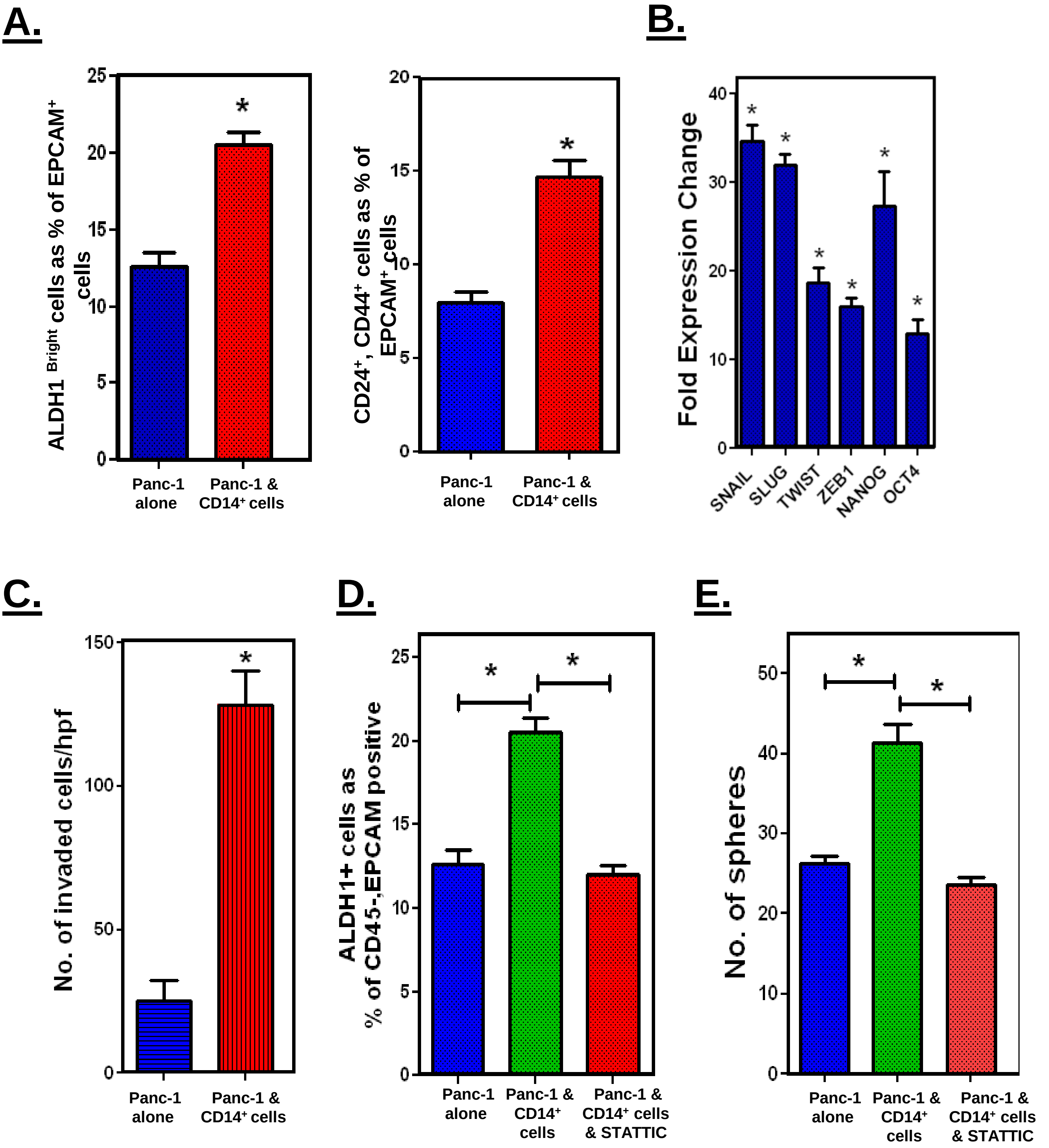

Supplementary figure 9:

A. $CD14^+$/HLA-$DR^{low/-}$ cells were co-cultured with Panc-1 for 72 hours and ALDH1, CD24 and CD44 staining was performed. Graph shows $ALDH1^{Bright}$ CSCs and $CD24^+$, $CD44^+$ cells as a percentage of $CD45^-$, $EpCAM^+$, PI^- cells.

B. Tumor EMT Markers: RT-PCR shows that markers of cell pluripotency and EMT were upregulated in Panc-1 tumor cells after co-culture with $CD14^+$/HLA-$DR^{low/-}$ cells.

C. Graph for invasion assays showing that Panc-1 tumor cells have increased invasion through matrigel matrix membrane in the presence of $CD14^+$ cells relative to tumor cells alone. Cells per high power field were quantified. Graph depicts the number of invaded cells per high powered field (mean ± S.E.M of three independent experiments).

D. Inhibition of STAT3 signaling in $CD14^+$/HLA-$DR^{low/-}$ cells prevents the increase in $ALDH1^{Bright}$ CSCs in Panc-1 from baseline. Representative flow-cytometry plots show $ALDH1^{Bright}$ CSC population (gated on $CD45^-$, $EpCAM^+$, PI^- cells) which is approximately 13.5% in Panc-1 alone, 20.8% when Panc-1 was co-cultured with $CD14^+$/HLA-$DR^{low/-}$ cells and 12.02% when Panc-1 was co-cultured with STATTIC treated $CD14^+$/HLA-$DR^{low/-}$ cells. Graph shows that inhibition of STAT3 signaling by STATTIC (20µM) blocks the increase in frequency of $ALDH1^{Bright}$ cells from baseline.

E. Bar graph shows tumor spheroid formation in Panc-1 cells with and without $CD14^+$/HLA-$DR^{low/-}$ cells in the co-culture. The mean number of tumor spheroids formatted after 10 days is depicted.

Panni et. al.
Supplementary Figure 10

pSTAT3 IN CD11b$^+$ CELLS AFTER CO-CULTURE WITH TUMOR CELLS (KCM)

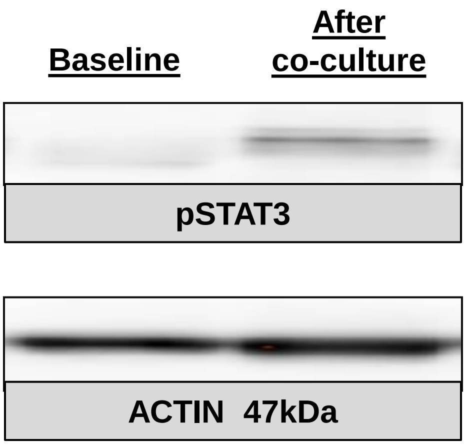

Supplementary Figure 10:

Western blot analysis shows increased phosphorylated STAT3 in $CD11b^+$ cells after co-culture with murine tumor cells (KCM).

Panni et. al.
Supplementary Figure 11

IL-6 LEVELS IN CD14+ CELLS WITH STATAT3 INHIBITION

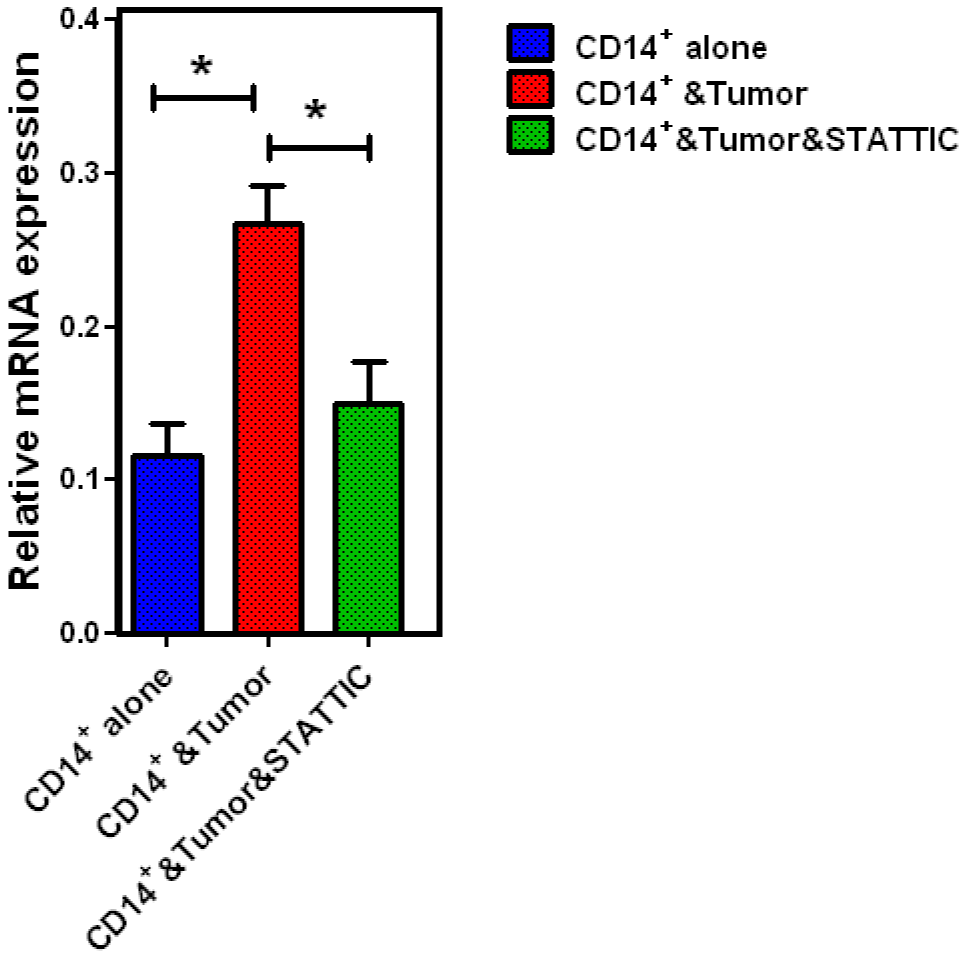

Supplementary Figure 11:

Inhibition of STAT3 signaling by STATTIC (20uM) downregulates the expression of IL-6 in tumor conditioned $CD14^{+}$/HLA-$DR^{low/-}$ cells by RT-PCR.

Bar graph depicts mean± SEM and asterisk (*) denotes statistically significant difference between the two groups $p<0.05$ by Mann-Whitney test.
